# Molecular evolution of the keratin associated protein gene family in mammals, role in the evolution of mammalian hair

**DOI:** 10.1186/1471-2148-8-241

**Published:** 2008-08-23

**Authors:** Dong-Dong Wu, David M Irwin, Ya-Ping Zhang

**Affiliations:** 1 State Key Laboratory of Genetic Resources and Evolution, Kunming Institute of Zoology, Chinese Academy of Sciences, Kunming, PR China; 2 Laboratory for Conservation and Utilization of Bio-resource, Yunnan University, Kunming 650091, PR China; 3 Graduate School of the Chinese Academy of Sciences, Beijing, PR China; 4 Department of Laboratory Medicine and Pathobiology, University of Toronto, Ontario, Canada; 5 Banting and Best Diabetes Centre, University of Toronto, Ontario, Canada

## Abstract

**Background:**

Hair is unique to mammals. Keratin associated proteins (KRTAPs), which contain two major groups: high/ultrahigh cysteine and high glycine-tyrosine, are one of the major components of hair and play essential roles in the formation of rigid and resistant hair shafts.

**Results:**

The KRTAP family was identified as being unique to mammals, and near-complete KRTAP gene repertoires for eight mammalian genomes were characterized in this study. An expanded KRTAP gene repertoire was found in rodents. Surprisingly, humans have a similar number of genes as other primates despite the relative hairlessness of humans. We identified several new subfamilies not previously reported in the high/ultrahigh cysteine KRTAP genes. Genes in many subfamilies of the high/ultrahigh cysteine KRTAP genes have evolved by concerted evolution with frequent gene conversion events, yielding a higher GC base content for these gene sequences. In contrast, the high glycine-tyrosine KRTAP genes have evolved more dynamically, with fewer gene conversion events and thus have a lower GC base content, possibly due to positive selection.

**Conclusion:**

Most of the subfamilies emerged early in the evolution of mammals, thus we propose that the mammalian ancestor should have a diverse KRTAP gene repertoire. We propose that hair content characteristics have evolved and diverged rapidly among mammals because of rapid divergent evolution of KRTAPs between species. In contrast, subfamilies of KRTAP genes have been homogenized within each species due to concerted evolution.

## Background

The availability of the rapidly increasing number of genome sequences provides opportunities for investigators to study evolutionary patterns that potentially account for morphological characteristics, and suggest the genetic basis for variation in phenotypes. In particular, gene families in which duplications, rate variation and pseudogenization occur frequently are likely involved in functional innovation and adaptation [1]. Examples of such gene families are those involved in the perception systems, for example, the odorant receptors [2-4], the vomeronasal receptors [5-8], and the sweet/umami and bitter receptors [9-12]. Furthermore, these studies should facilitate our understanding of the general evolutionary trends in genomic complexity and lineage-specific adaptation [1]. Here, we studied the evolutionary patterns of the keratin associated protein (KRTAP) gene family, whose encoded proteins are major components of hair, with the goal of revealing the underlying basis for unique mammalian hair and its phenotypic diversity.

Hair is a unique character found on all mammals, but not on other animals, where it plays a crucial role in the retention of heat within these homoiotherms and presumably contributed significantly to the rapid radiation of mammals and their rise to become the dominant terrestrial vertebrate [13]. Other functions of hair include sexual dimorphism, attraction of mates, and protection of skin [14]. An interesting event in hair evolution has been its loss in humans [15,16], however; humans actually have a similar density of hair follicles to that seen in apes [15]. Comparative studies have concluded that hair presents similar structure and modality of growth throughout mammals [17-19]. For example, the overall ultrastructure of hair and the distribution of keratins in monotremes are similar to that of marsupial and placental mammals [18], and the localization of acidic and basic keratins in marsupial hairs is similar to that in placentals [19]. However, most studies have focused on keratins rather than the keratin associated proteins.

The major components of hair are alpha-keratins and keratin associated proteins, each of which are encoded by multigene families. The alpha-keratins include two multigene subfamilies, type I (acidic) and type II (basic) [20,21], and form the intermediate filament cytoskeleton of all epithelia providing stability against stress [22]. In humans, the alpha-keratin gene family has been extensively studied demonstrating that there are 54 functional genes that are clustered on chromosomes 12q13.13 and 17q21.2 that show differing expression patterns during hair development [23]. Hair keratins form an intermediate filament (IF) network by co-polymerization of type I and type II members, in trichocytes, which are cells that populate the central hair-forming compartment of the anagen hair follicle [24,25]. In the hair cortex, hair keratins IFs are embedded in an in interfilamentous matrix, which consists of hair keratin-associated proteins (KATAP, usually abbreviated as KAP) [26]. KRTAP contains two major groups: high/ultrahigh cysteine (HS) and high glycine-tyrosine (HGT) that are considered to have originated independently, and are essential for the formation of rigid and resistant hair shafts through their extensive disulfide bond cross-linking with the abundant cysteine residues of hair keratins or hydrophobic interactions with keratins [23,26-28]. The genes have been grouped into 27 subfamilies, termed KRTAP1 to KRTAP27, based upon phylogeny [23,29]. In humans, about 100 KRTAP genes are identified, that are in five tandemly arranged clusters (chromosomal regions 11p15.5, 11q13.4, 17q21.2, 21q22.1, and 21q22.3) [30-34]. Previous research on KRTAP genes has focused on their function and expression, with little emphasis on the origin and evolution of this gene family. Here, we investigate the evolution of the KRTAP gene family, including phylogeny and classification, and the mechanisms involved, such as gene duplication, gene conversion, in our endeavor to resolve hair's evolutionary history and to explain the diversity observed in extant mammals. We find different repertoires among mammals which potentially explain the differing hair features of different lineages. An expanded KRTAP gene repertoire was found in rodents. Surprisingly, despite the lack of hair, human had a similar number of genes with other primates. Large-scale gene conversion events were detected in high cysteine KRTAP but fewer in high glycine/tyrosine, and the latter genes evolve more dynamically. Compared to the conserved structure and modality of keratins within mammals, the significant divergence of characteristics of hair among placental, marsupial and monotreme species is likely due to interspecific divergence of KRTAP sequences.

## Results

### Inventory of KRTAP genes in mammals

The recent rapid increase in the availability of comparative genomic data is facilitating the illumination of evolutionary features of organisms. Particularly, mammals are well represented with data from: placental mammals including primates – human [35], chimpanzee [36] and rhesus macaque [37]; rodents – mouse [38] and rat [39]; carnivore – dog [40]; a marsupial, the opossum [41]; and a monotreme, the duck-billed platypus [42].

Some gene sequences in GenBank http://www.ncbi.nlm.nih.gov have been submitted independently by several researchers. KRTAP genes have been shown to have size polymorphism within populations [43-45], and sequences from different individuals at these loci are diverse and may not align well. For instance, we identified that a KRTAP gene cluster in Contig: NT_113931 actually corresponds to the KRTAP region on Chr17 of the human genome, except that it differs in sequence length for several KRTAP genes. Therefore, we removed the genes from Contig: NT_113931 from our analyses. Furthermore, since many of the draft genome sequences were generated by whole genome shotgun assembly, we took care to identify redundant KRTAP sequences and excluded these from our analysis.

Since KRTAP genes are clustered as tandem arrays at just a few chromosomal locations and the genome sequences have high coverage (except platypus), we should be able to obtain near-complete and non-redundant KRTAP gene inventories from these mammalian genomes after in-depth screening and examination (summarized in additional file 1). In contrast to the mammalian genomes, we were unable to identify any KRTAP-like sequences from the chicken, lizard, *Xenopus tropicalis *or zebrafish genomes. We also note that the keratin associated protein 10-4 annotated in the chicken genome in GenBank (gene id: 425968) is actually an oncogenic transcription factor (JAC), and is not KRTAP-like nor has homology with any mammalian KRTAP sequences. As an alternative strategy to identify KRTAP genes in the chicken, we used Mapviewer from NCBI http://www.ncbi.nlm.nih.gov/mapview/ to identify genes flanking the KRTAP gene clusters in mammals and searched the chicken genome using BLAST to identify orthologous genomic regions. While conserved synteny of the flanking genes was observed within the chicken genome, KRTAP-like genes could not be found. These results indicate that the KRTAP gene clusters are unique to mammals and have been inserted into in the ancestral mammalian genome.

**Figure 1:**
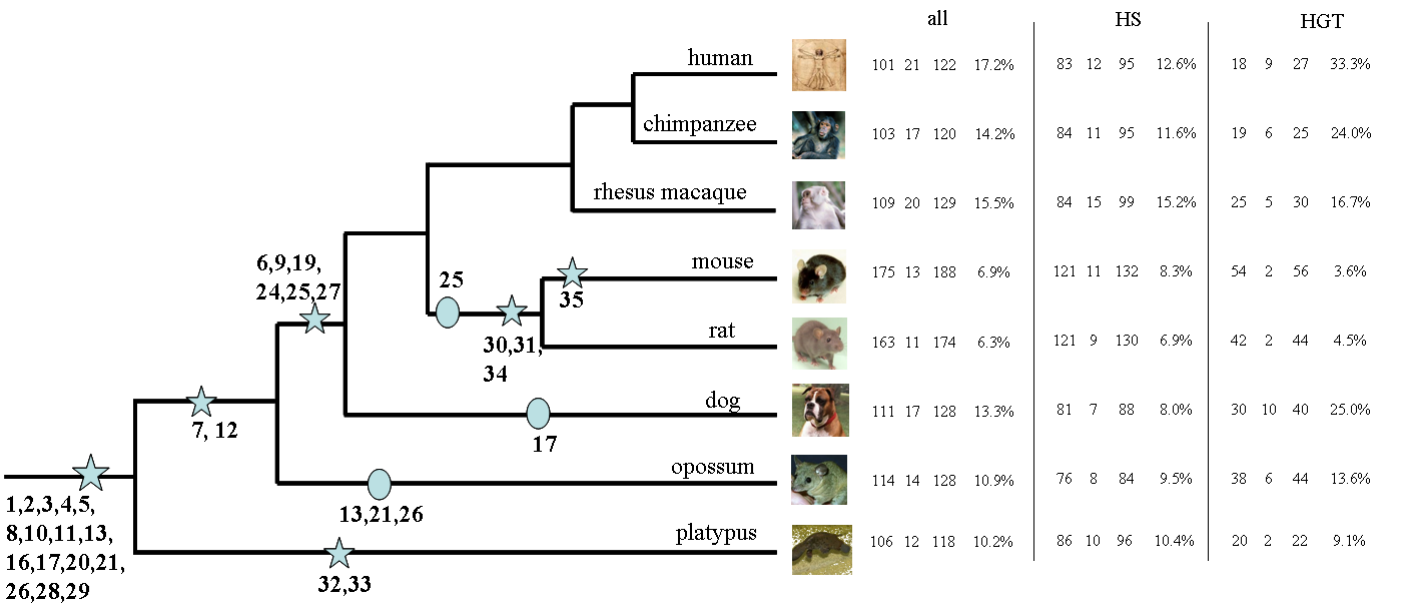
**Summary of the evolution of the KRTAP gene repertoires in eight mammals.** Major events in the evolution of the KRTAP gene family are summarized. The number of KRTAP genes in each species is indicated on the right with "all", "HS", and "HGT" indicating patterns for all, high/ultrahigh cysteine, and high glycine-tyrosine KRTAPs, respectively. The columns represent the numbers of intact, interrupted and all genes and the percentage of pseudogenization calculated as the ratio of number of disrupted gene to all genes, for each of the categories of genes. Stars in the phylogeny indicate the origin of subfamilies, while circles indicate losses.

We classified our identified KRTAP gene sequences into two categories, intact and pseudo-genes. Pseudogenes are defined as coding sequences that are disrupted by frameshifts and/or stop codons. In placental mammals, rodents appear to contain an expanded KRTAP gene repertoire with a lower level of pseudogenization relative to other mammals both in high (including ultrahigh) cysteine and high glycine KRTAPs (fig. 1). Surprisingly, although humans have the lowest number of intact genes (101) and the highest number of pseudogenes (21, 17.2% of total number), the complete repertoire (122) is still similar in number to that of other mammals. Potentially, changes in the levels of expression of KRTAP genes may account for the relative hairlessness of humans. Opossum has a slightly larger repertoire of genes, and the repertoire in platypus is probably much larger than that we reported, since a more complete genome sequence is required to accurately define gene number.

### Classification of KRTAP gene family, and tandem cluster in the genomes

**Figure 2:**
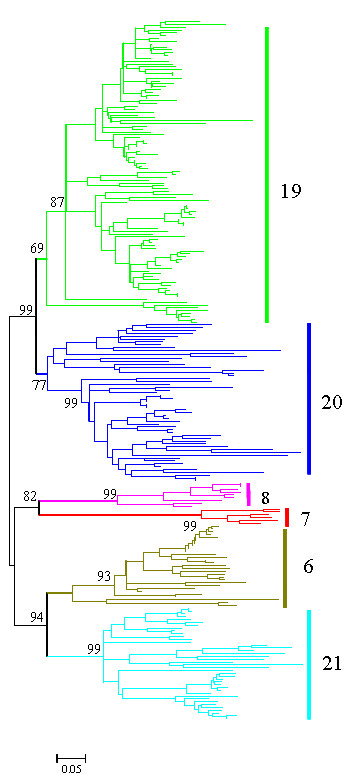
**Phylogenetic tree of high glycine-tyrosine KRTAP genes of all mammals.** Simplified phylogeny of all of the high glycine-tyrosine KRTAP genes generated by the neighbor-joining algorithm using p-distances. Genes of each subfamily are represented by different colors. Numbers on branches are the reliabilities of the branches which are calculated by interior branch tests with 1,000 replications. The bars indicate six subfamilies (6, 7, 8, 19, 20 and 21) of HGT-KRTAP genes.

Without the influence of evolutionary constraint, pseudogenes evolve faster then functional genes. Accordingly, substitution bias could generate errors in constructing phylogenies; therefore all pseudogenes were excluded from our preliminary analysis on subfamily classification. The high levels of divergence between species and/or subgroups and the homogenization within them caused by frequent gene conversion (see following text), may also introduce a bias into the construction of a phylogeny of all of the high cysteine KRTAPs. To circumvent this problem, we first constructed a neighbor-joining tree of just the human high cysteine KRTAP protein sequences, and used this phylogeny to identify subfamilies (Additional file 2: figure 1). The majority of genes for each subfamily were found as subfamily-specific tandem cluster in the genome (Additional file 2: figure 1 and Additional file 1). Genes with close relationships in our phylogeny tended to be most closely linked in the genome. This positive correlation between phylogeny and chromosomal location has previously been observed in several other gene families [46,47]. Thus we used chromosomal location in combination with phylogeny to refine our subfamily classification. In addition we also considered amino acid sequence composition to generate our final classification. Next we constructed phylogenies of all of the high cysteine KRTAPs for each species alone (Additional file 2: figures 2–8), or combined with the human sequences (Additional file 2: figures 9–15), as well as a phylogeny of the combined mouse and rat sequences (Additional file 2: figure 16). From these phylogenies, together with chromosomal location and amino acid composition, we could identify several new subfamilies not reported previously, which we name subfamilies 28–35. Additionally, we grouped the previously characterized subfamilies 14 and 15 into subfamily 13 based on our phylogeny. In a similar manner, we grouped the glycine-tyrosine rich KRTAPs into 6 subfamilies (subfamilies 6, 7, 8, 19, 20 and 21) based on the phylogenetic tree (fig. 2, and Additional file 2: figure 17). The previously defined human subfamily 22 was combined with subfamily 19. Our recommendations have been endorsed by the HUGO Gene Nomenclature Committee [48].

**Figure 3:**
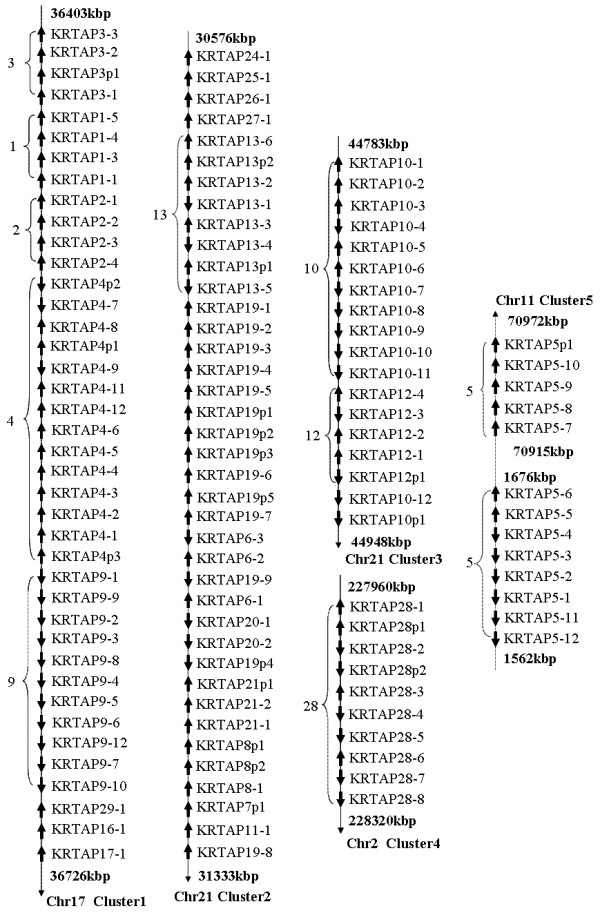
**Summary of the chromosomal distribution of KRTAP genes in the human genomes.** The relative genomic location of each KRTAP gene is shown for chromosomes 2, 11, 17 and 21. Each gene, and distances between genes are not to scale. Arrowheads indicate the direction of transcription. The clusters are also labeled.

Our phylogeny of intact genes indicated that genes within subfamilies are clustered in the genomic location (fig. 3, and Additional file 1). When pseudogenes were included in the phylogenetic analysis they were also found to be most closely related to their genomic neighbors. To classify interrupted genes that were annotated in unmapped (i.e., unassembled) genomic locations, BLAST was performed against the non-redundant database in GenBank to identify the best hit KRTAP genes, and phylogenetic classification of these were used to help classify the pseudogenes.

It should be noted that there is typically only about one gene per species for subfamilies 16, 24–27, and 29 (table 1), thus, these subfamilies were not used for our subsequent analysis for gene conversion.

**Table 1:** Numbers of KRTAP gene in each subfamily in eight mammalian species.

	subfamily	human	chimpanzee	rhesus macaque	mouse	rat	dog	opossum	platypus
HS-KRTAP									
	1	4(0)	4(0)	4(0)	4(0)	4(0)	4(0)	4(0)	4(1)
	2	4(0)	4(0)	4(1)	4(0)	4(0)	4(0)	4(0)	4(0)
	3	4(1)	4(0)	4(1)	4(0)	4(0)	4(0)	4(0)	5(0)
	4	14(3)	14(3)	14(5)	18(2)	16(3)	8(0)	30(6)	19(3)
	5	14(2)	15(2)	18(1)	18(0)	20(1)	14(2)	9(0)	7(1)
	9	11(0)	11(1)	9(2)	7(2)	7(2)	7(1)	0	0
	10	13(1)	13(1)	13(0)	14(0)	14(0)	14(0)	13(0)	12(0)
	11	1(0)	1(0)	1(0)	1(0)	1(0)	1(0)	1(0)	1(0)
	12	5(1)	5(1)	5(1)	5(0)	6(0)	7(0)	6(2)	0
	13	8(2)	6(2)	9(3)	14(4)	13(3)	9(3)	0	5(0)
	16	1(0)	1(0)	1(0)	1(0)	1(0)	1(0)	1(0)	1(0)
	17	1(0)	1(0)	1(0)	1(0)	1(0)	0	1(0)	1(0)
	24	1(0)	1(0)	1(0)	1(0)	1(0)	1(0)	0	0
	25	1(0)	1(0)	1(1)	0	0	1(0)	0	0
	26	1(0)	1(0)	2(0)	1(0)	1(0)	1(0)	0	3(0)
	27	1(0)	1(0)	1(0)	1(0)	1(0)	1(0)	0	0
	28	10(2)	11(1)	10(0)	13(0)	12(0)	10(0)	10(0)	9(0)
	29	1(0)	1(0)	1(0)	1(0)	1(0)	1(0)	1(0)	1(0)
	30	0	0	0	19(2)	19(0)	0	0	0
	31	0	0	0	3(1)	3(0)	0	0	0
	32	0	0	0	0	0	0	0	7(3)
	33	0	0	0	0	0	0	0	17(2)
	34	0	0	0	1(0)	1(0)	0	0	0
	35	0	0	0	1(0)	0	0	0	0

HGT-KRTAP									
	6	3(0)	3(0)	5(1)	6(0)	6(0)	6(0)	0	0
	7	1(1)	1(0)	1(0)	1(0)	1(0)	1(0)	1(0)	0
	8	3(2)	3(2)	1(0)	1(0)	1(0)	1(0)	1(0)	2(0)
	19	14(5)	10(3)	15(4)	12(1)	12(1)	14(6)	0	0
	20	2(0)	2(0)	3(0)	32(0)	21(1)	8(4)	42(6)	9(1)
	21	4(1)	6(0)	5(0)	4(1)	3(0)	10(0)	0	11(1)

The number in parentheses indicates the number of disrupted genes. HS-KRTAP and HGT-KRTAP indicate high cysteine and high glycine/tyrosine KRTAP respectively.

### Chromosome distribution of KRTAP

KRTAP genes are distributed mainly at five genomic regions in placental and marsupial genomes: Cluster 1 contains genes from subfamilies KRTAP 1, 2, 3, 4, 9, 17, 16, and 29. Cluster 2 contains genes from subfamilies 13, 24–27 and all glycine-tyrosine rich KRTAPs. Cluster 3 possesses genes from subfamilies 10 and 12. Cluster 4 encodes genes of subfamily 28. Cluster 5 corresponds to genes of subfamily 5 (fig. 3). Some variation in gene distribution is observed in some species. In rodents, the new subfamilies 30 and 31 have been inserted into the genomic locations of subfamilies 4 and 9 respectively. Paralogous KRTAP gene clusters have been mapped to human chromosomes 11q13 and chr11p15, with genes residing in these two clusters intermingled in the phylogeny (fig. 3.). Thus suggests that the KRTAP gene cluster at 11q13 is derived from 11p15 potentially representing a segmental duplication. The new chromosome 11q13 gene cluster is unique to primates as an orthologous region is also found in the chimpanzee and rhesus macaque genomes but not in others mammals (additional file 1). In a similar manner the dog genome has generated a new cluster on chromosome 31 that includes three genes, one from subfamily 10 and two from subfamily 12. Similar mechanisms for the origin of new genes at new genomic locations have been observed for other gene families [49,50].

### Amino acid composition comparison of KRTAP subfamilies

Previous research has classified the keratin associated proteins by their amino acid composition into three major groups: high-sulfur (~16–30% cysteine), ultra-high sulfur (> 30% cysteine), and high-glycine/tyrosine [26]. Subfamilies 1, 2, 3, 10, 12, 16, 29 and 31, belong to the high-sulfur group; subfamilies 4, 5, 9, 17, 28, 30, 32 and 33 are ultra-high sulfur (table 2). Many high cysteine genes also have a high content of serine. Interestingly, subfamilies 11, 13, 24–27, 29, 34 and 35 have high serine content but relative low cysteine (table 2). The newly identified subfamilies 28 and 30, for which there is no functional or expression data, have the highest cysteine content (39.5%, 50.1% respectively).

**Table 2:** Amino acid composition of KRTAPs subfamily genes in mammals.

HS-KRTAP	subfamily	C	G	L	P	Q	S	T	Y
	1	26.57	10.04	1.67	9.22	6.84	15.55	8.04	1.88
	2	29.1	4.42	1.44	14.4	6.03	10.33	9.25	0.56
	3	19.93	4.72	8.01	15.6	2.9	8.53	10.84	1.53
	4	37.37	2.98	1	10.57	5.47	16.21	7.91	0.51
	5	35.86	23.67	0.1	5.01	3.39	19.25	0.65	0.27
	9	35.26	2.65	1.02	11.04	7.17	12.74	13.95	1.32
	10	27.61	2.55	3.34	13	6.1	18.76	4.62	0.52
	11	13.1	8.08	4.08	8.24	7.45	14.75	12.16	2.43
	12	22.86	2.57	2.17	13.81	6.18	21.12	4.02	1.33
	13	11.47	10.61	5.73	7.41	4.17	21.31	5.88	7.49
	17	36.06	31.44	0.26	4.49	4.23	9.91	3.17	0
	24	9.77	5.05	7.45	9.38	3.99	17.55	7.31	7.18
	25	7	5.07	5.56	8.21	6.28	19.08	3.86	6.28
	26	11.3	9.04	8.47	11.45	4.01	18.08	5.14	3.24
	27	8.71	4.07	6.59	7.81	7.81	18.06	7.73	1.38
	28	39.54	33.66	0.01	1.6	3.93	5.8	2.69	1.02
	29	16.27	6.01	3.34	11.42	8.33	16.7	7.84	2.39
	30	19.35	2.07	2.43	14.56	5.07	17.12	6.19	2.15
	31	26.53	1.02	3.06	11.9	4.76	15.08	10.66	0.11
	32	38.72	3.4	1.91	16.17	4.26	10	8.94	0.21
	33	32.18	5.42	2.65	15.29	1.52	9.17	3.19	0.11
	34	50.73	4.65	0.17	9.54	11.11	4.42	8.55	0.23
	35	9.19	8.38	4.32	11.08	4.05	21.62	8.11	5.68
	36	10.34	4.6	8.05	11.49	6.9	20.69	6.9	1.15

HGT-KRTAP	6	13.61	40.26	4.87	0.19	0.05	7.59	0.29	22.87
	7	8.81	19.16	4.79	7.28	0.19	11.49	6.13	12.26
	8	5.72	23.97	3.94	7.16	0	8.59	2.15	20.04
	19	6.07	36.52	4.22	1.38	0.33	10.8	0.33	19.96
	20	13.9	37.61	4.61	1.73	0.26	5.72	0.23	24.2
	21	17.2	36.82	0.93	1.03	0.24	12.6	0.87	20.98

The average amino acid content (%) of high cysteine (HS) and high glycine/tyrosine (HGT) KRTAP subfamily genes is shown. C (cysteine), G (glycine), L (leucine), P (proine), Q (glutamine), S (serine), T (threonine) and Y (tyrosine) are single letter codes for amino acids that are abundant in some KRTAP proteins.

### Concerted evolution yields a high GC (Guanine and Cytosine) content in high cysteine KRTAP gene family

The evolutionary patterns observed in a multigene family can be attributed to two traditional models: concerted evolution and the birth-and-death process [47]. Members of a gene family under concerted evolution evolve in a concerted manner rather than independently, as a mutation occurring in one member will spread through the entire gene family by the repeated occurrence of unequal crossover and/or gene conversion [47]. Concerted evolution therefore results in the distance between pairs of genes remaining low. Considering the abundant species-specific clusters in the KRTAP gene phylogeny, many should be candidates for concerted evolution. Gene conversion plays a parallel role to unequal crossing over, with their major difference being that the latter can change the copy number of a gene; however, it is difficult to distinguish between these two mechanisms.

We identified potential gene conversion events within each subfamily using the methods implemented in the GeneConv program [51] which identifies identical fragments shared between pairs of nucleotide sequences. We found a large number of KRTAP gene pairs for which gene conversion events are suggested in the high/ultrahigh cysteine subfamilies, but significantly fewer, only 4 pairs, in high glycine-tyrosine subfamilies (χ^2 ^= 73.85, p << 10^-10^) (summarized in additional file 3). The distribution of gene conversion events differs between species, suggesting that different levels of gene conversion occur in each species. For example, 32 gene pairs in rat subfamily 10 suggested evidence for gene conversion, but only 7 pairs were identified for this subfamily in the mouse. The RDP2 program also detected a large number of recombination events within the high cysteine KRTAP genes but fewer in the high glycine/tyrosine genes (additional file 4), and typically suggested that different gene pairs were involved. The identification of different gene pairs by the two methods may reflect differences in how the two programs identify recombination events.

**Figure 4:**
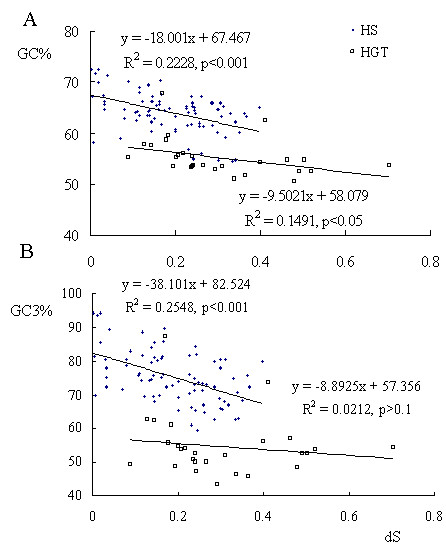
**Correlation between GC (Guanine and Cytosine) content and divergence between KRTAP genes.** The synonymous substitution rates (dS) among paralogs within each subfamily and codon GC content (GC%) (A) and third codon GC content (GC3%) (B) of each subfamily is plotted. Dots and circles represent high cysteine KRTAP (HS) and high glycine-tyrosine KRTAP (HGT), respectively. The linear regression formulae for GC and dS are shown.

Evidence for concerted evolution was also found in changes in base composition of the genes. Gene conversion is a nonreciprocal recombination process in which a DNA segment of a recipient gene is copied from a donor gene and occurs during the repair of double strand breaks by recombination [52]. Recently, it has been discovered that gene conversion introduces a GC nucleotide bias into sequences, the biased gene conversion (BCG) concept, resulting in the enrichment of GC content in DNA sequences undergoing concerted evolution [52,53] resulting in a positive correlation between rate of gene conversion and GC content. Therefore we examined the GC content of KRTAP coding sequences, which we propose scales with the rate of gene conversion. To evaluate the contribution of concerted evolution, we also scaled the level of gene conversion by distance between paralogs in each subfamily. It has been reported that the frequency of gene conversion correlates negatively with the divergence between gene pairs [54]. Synonymous nucleotide sites are expected to evolve neutrally and can be used to evaluate the relative evolutionary divergence between pairs of genes. Intriguingly, high cysteine KRTAP genes contain extremely high GC content, and the GC content is negatively correlated with the divergence within the high cysteine KRTAP gene subfamilies (fig. 4). Comparatively, high glycine/tyrosine KRTAP contain relatively lower GC content (~50%), and a higher synonymous substitution rate (fig. 4), consistent with the detection of fewer gene conversion events.

Phylogenetic relationships can also suggest sequence homogenization, and potentially uncover the level of gene conversion. In the tree of human and chimpanzee KRTAP protein sequences, large quantities of one-to-one orthologies were identified (additional file 2: figure 9), suggesting that intraspecific gene conversion in the past 5 MYRs ago has not obscured orthologous relationships of the human and chimpanzee genes. The number of these one-to-one orthologies is reduced as phylogenetic distances between species pairs increases (additional file 2: figures 9–15).

### More dynamical evolution of high glycine/tyrosine KRTAPs

**Figure 5:**
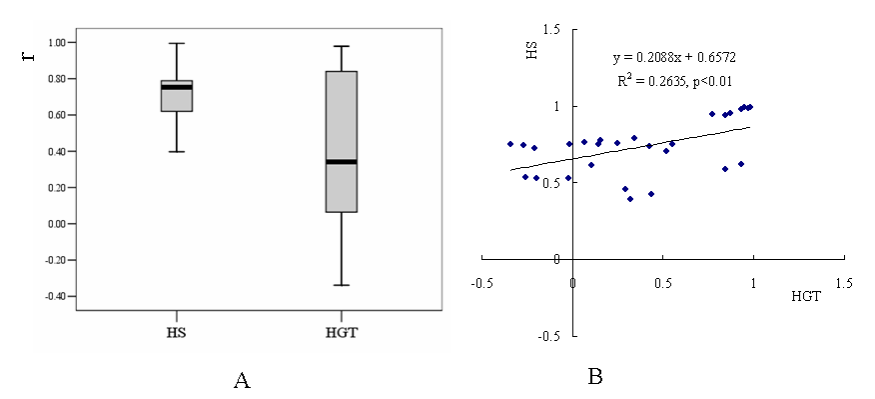
**Evolutionary dynamics of KRTAP genes.** A comparison of the evolutionary dynamics as evaluated by Pearson correlation coefficient (r) of gene numbers of each subfamily between species is shown. A) The coefficient value for the high cysteine KRTAP and high glycine/tyrosine genes is shown with standard errors of the estimates. The correlation coefficient of the high cysteine genes is significantly higher than for the high glycine/tyrosine genes. B) A linear regression of the coefficients between high cysteine KRTAP and high glycine/tyrosine KRTAP genes. The two groups of coefficient values are positively correlated at the 1% level.

Higher GC content in high cysteine KRTAP compared to high glycine/tyrosine genes reveals that strong concerted evolution is occurring in former, and that they evolve in a more stably manner. Accordingly, we compared the evolutionary dynamics of the two kinds of KRTAPs by calculating the Pearson correlation coefficient of the number of genes within each subfamily between species. As expected, the coefficient value is significantly higher for high cysteine KRTAP than for high glycine/tyrosine genes (p < 10^-4 ^by Wilcoxon signed ranks test) (fig. 5A), and the two groups of coefficient values are positively correlated (fig. 5B). In particular, subfamily 20 evolved with a very dramatic variation in gene numbers, e.g. mouse and opossum have 30 and 42 genes respectively, but humans only have 2. As the number of gene conversion events detected was close to zero, the false positive rate for detecting positive selection in subfamily 20 should be very low. Positive selection, which was detected on the rat and opossum lineages by likelihood ratio tests (table 3), thus is proposed to be the major force for the dynamic evolution of subfamily 20 and possibly for other high glycine/tyrosine KRTAP genes.

**Table 3:** Likelihood ratio tests for positive selection in subfamily 20 by the site-specific models M1a vs M2a within mouse, rat, and opossum.

	2ΔL	d.f.	Parameters estimated under positive selection model	p-value
mouse	2.819	2	p_0 _= 0.686, p_1 _= 0.000, p_2 _= 0.314 ω_0 _= 0.107, ω_1 _= 1.000, ω_2 _= 1.480	0.244
rat	13.910	2	p_0 _= 0.657, p_1 _= 0.280, p_2 _= 0.064, ω_0 _= 0.108, ω_1 _= 1.000, ω_2 _= 13.370	9.54E-04
opossum	18.575	2	p_0 _= 0.535, p_1 _= 0.411, p_2 _= 0.054 ω_0 _= 0.096, ω_1 _= 1.000, ω_2 _= 6.894	9.26E-05

## Discussion

We have described the near-complete inventories of KRTAP genes from the genomes of human, chimpanzee, and rhesus macaque representing primates, mouse and rat representing rodents, dog representing carnivore, opossum representing marsupial, and platypus as a monotreme, and conducted comprehensive analyses of the evolutionary patterns for this gene family, including phylogenetic classification, and the detection of gene conversion.

Among the mammals that we studied, the genomes of each species have members of most of the subfamilies suggesting that the majority of KRTAP gene subfamilies originated and diverged before the mammalian radiation (fig. 1). Accordingly, the mammalian ancestor should also have had a high diversity of KRTAP genes and enjoyed a similar range and spectrum of hair characteristics that is seen in modern mammalian species. The genes for subfamilies that emerged in the early mammals account for about 74% total number of genes in present mammalian genomes. We advocate that the rapid emergence of the KRTAP gene family correlates with the evolution of mammalian hair and that the rapid emergence of plentiful hair contributed to the successful radiation of homothermal mammals by helping them to retain body heat since hair acts as an insulator [13]. In contrast, *Homo sapiens *has recently lost body hair function, presumably because humans can obtain heat and can keep cold out by using clothing. Despite a lack of hair, humans actually have a similar density of hair follicles to apes [15], which possibly explains why human do not have a significant fewer number of KRTAP genes. Perhaps, the changes in human hair are due to the reduction in expression of the KRTAP genes. In contrast to humans, rodents have an expanded KRTAP gene family. Perhaps, mouse and rat need more hair as they are adapted to the nocturnal environment. Despite the proposed similar range and diversity of hair characteristics in the ancestor of mammals, the current traits, and content, of hair likely diverged significantly from their ancestral characteristics as KRTAP sequences have diverged dramatically among species due to gene conversion.

Both gene conversion and unequal crossing over have occurred during the evolution of the KRTAP. The evolution of several KRTAP gene subfamilies, such as subfamilies 1, 2 and 3 fit well with a model of punctuated equilibrium (table 1), where morphological divergence as well as speciation occurs in a burst-like manner with rapid evolutionary change followed by long periods of constancy [55]. These KRTAP genes show evidence for fewer gene duplication or loss events, suggesting that a low level of unequal crossing over has occurred within these subfamilies, and that gene conversion may predominate. In addition, the syntenic arrangement of genes of these subfamilies has not been disturbed by post-duplication rearrangement events, suggesting a strong constraint has been exerted upon their distribution. In contrast, other KRTAP genes, such as subfamilies 4 and 5 (table 1) have evolved relatively dynamically likely including unequal crossing over as changes in the copy number of this subfamily is observed between species.

Changes in gene numbers within subfamilies between mammals may explain differences in the observed hair morphology between species. No genes of subfamily 9 were identified in opossum or platypus, suggesting that this subfamily originated after the divergence of placental and marsupial mammals. In a similar manner, genes of subfamily 12 emerged before the divergence of marsupials but after the divergence from monotremes (fig. 1). Intriguingly, subfamily 13 has been lost on the marsupial lineage, and the subfamilies 24, 26 and 27 which are adjacent to subfamily 13 in the genome, are also not found in the opossum genome. We used Mapviewer from NCBI to identify genes flanking this cluster in the human genome and searched the opossum genome using BLAST to identify an orthologous genomic region. An ortholog of the human gene that flanks the human KRTAP genes is only about 13 kbp away from the remainder of the KRTAP cluster in the opossum genome, which indicates the loss of this region in the opossum was due to a deletion event. Subfamily 30, which is a member of the ultra-high sulfur (~50%) group, along with subfamily 34 are unique to rodents, thus may be partially responsible for the unique characteristics of hair in rodents. Subfamily 35 is mouse-specific, while subfamilies 32 and 33 are platypus-specific, which could account for species-specific hair characteristics.

The high-glycine/tyrosine (HGT) gene repertoire evolve more dynamically within mammals with increased levels of pseudogenization (fig. 1), for example, mice possesses 56 genes, while primates have considerably fewer genes with only 27 and 25 genes in human and chimpanzee respectively. The gene number in platypus is likely to be underestimated because HGT cluster in platypus is shorter than that of other mammals (only about 100 kbp in platypus, but ~500 kbp in human, ~800 kbp in mouse, and ~350 kbp in opossum), and the incomplete nature of this genome sequence. However, the dynamics of HGT subfamilies does not appear to be due to unequal crossing over, as unequal crossing over should also generate a GC content bias that is not observed in these genes (personal communication, Gabriel Marais).

Tandemly arrayed paralogous genes with similar function can provide combinatorial complexity to biological diversity [56]. This extraordinary evolutionary feature has been observed in many multigene families responsible for processes that face enormous external signals. For instance, the sensory system such as the olfactory receptor, vomeronasal receptor and sweet/umami receptor, bitter receptor genes, require a huge combination of diverse receptors for the diverse ligands they encounter in a tremendous range of molecular environments [57]. Another classic example is the immunoglobulin and T-cell receptor protein superfamily which use recombination to generate large quantities of antigen recognition complexes to allow an immune responses to rapidly evolving pathogens [58]. We therefore speculate that an analogous process occurs within the KRTAP gene family, where the KRTAP and keratin proteins have combined in unique combinations to generate the high diversity of hair phenotypes that are observed both between and within species, and even within individuals.

## Conclusion

We have described the near-complete inventories of KRTAP genes in eight mammalian genomes. We found that the KRTAP family was unique to mammals, KRTAP gene repertoire was expanded in rodents, and surprisingly, humans had a similar number of genes as other primates, inconsistent with the hairlessness of humans. The high glycine-tyrosine KRTAP genes have evolved more dynamically, with fewer gene conversion events and thus have a lower GC content compared with high cysteine KRTAPs. We propose that the mammalian ancestor should have a diverse KRTAP gene repertoire, and that hair content characteristics have evolved and diverged rapidly among mammals because of rapid divergent evolution of KRTAPs between species caused by concerted evolution.

## Methods

### Retrieval of sequences

Some KRTAP genes have previously been annotated to possess introns, which generally complicates gene prediction and identification. All of the intron-containing KRTAP genes have short introns, and the sequences of these introns are similar to the repeated regions found within the exons, and many are predicted to be alternatively spiced. All of the introns could be included in a primary transcript that can be translated in-frame with the coding exons, they just have longer repetitive regions. Therefore, we hypothesize that all of the KRTAP genes can generate an mRNA sequence that is intron-less.

We identified KRTAP gene repertoires in the genome assemblies from human (*Homo sapiens*) (build36.2), chimpanzee (*Pan troglodytes*) (build2.1), rhesus macaque (*Macaca mulatta*) (build1.1), mouse (*Mus musculus*) (build36.1), rat (*Rattus norvegicus*) (RGSC v3.4), dog (*Canis familiaris*) (build2.1), opossum (*Monodelphis domesticus*) (MonDom5) and platypus (*Ornithorhynchus anatinus*) (build1.1). We used the BLASTn algorithm [59] to search these genomes using all known human KRTAP genes as queries. Each newly identified putative KRTAP gene was used as a query using BLAST http://blast.ncbi.nlm.nih.gov/Blast.cgi against the non-redundant GenBank database to check whether their best hit was a KRTAP gene. The chicken (*Gallus gallus*) (Build 2.1), zebrafish (*Danio rerio*) genomes (Zv6) in NCBI [60], lizard (*Anolis carolinensis*) genome at UCSC http://genome.ucsc.edu/ and the *Xenopus tropicalis *genome at JGI [61] genomes were also searched using BLAST for KRTAP-like sequences. Sequences that possessed an interrupting stop codons and/or frame-shifts caused by insertions or deletions were denoted as pseudogenes.

### Phylogenetic reconstruction

In order to classify the members of the KRTAP gene family, protein sequences were used to construct phylogenetic trees using the neighbor-joining method with p-distances with MEGA3.0 http://www.megasoftware.net[62] after alignment with ClustalW http://www.ebi.ac.uk/Tools/clustalw/index.html[63]. The reliability of the trees was evaluated by the interior branch tests with 1,000 replications.

### Detection of recombination

We employed the GeneConv program http://www.math.wustl.edu/~sawyer/geneconv/[51] to identify potential gene conversion events in the KRTAP coding sequences. Gene conversion is a process where a segment of DNA from one allele of a gene is copied and replaces the sequence in another allele or gene. Accordingly, GeneConv extends a method previously described by [51] and detects this process by identifying shared fragments between pairs of sequences. Global Bonferroni corrected P values were calculated to evaluate the statistical significance of the observed fragment lengths and are compared to a simulated distribution (10,000 iterations) of the same number of sequences with similar variation. Lower P values suggest a greater probability that a gene conversion event has occurred. GeneConv has a significant limitation in that it is unable to distinguish between gene conversion and unequal crossing over events, but to date no other effective bioinformatic method have been described that can distinguish between these two types of events. We therefore, can not distinguish gene conversion and unequal crossing over.

The divergence among paralogs could also reflect the level of conversion which can homogenize paralogs. Nucleotide sequences were back-translated from protein sequences after alignment by ClustalW [63]. Alignments were modified manually if necessary. Approximate synonymous substitution rate (Ks) values within subfamilies were calculated by the modified Nei-Gojobori (p-distance) method with a transition/transversion ratio of 2 [64].

Recombination events were also detected using the RDP2 software package http://darwin.uvigo.es/rdp/rdp.html[65]. Evidence for recombination was detected by running RDP, BootScan, MaxChi and Chimaera with 1,000 permutations. Sequences were considered linear. The highest acceptable P value cut-off was set to 0.01. Bonferroni correction was employed.

### Adaptive evolution analysis

The site-specific models M1a and M2a implemented in PAML http://abacus.gene.ucl.ac.uk/software/paml.html were used to detect potentially positively selected sites in the subfamily [66-68]. Considering the high false positive rate of likelihood ratio tests, particularly when there is frequent recombination [69,70], we only detected positive selection in subfamily 20, a family in which gene conversion does not appear to occur and has expanded dramatically on the mouse, rat and opossum lineages.

### Statistical analysis

We calculated the potential number of gene pairs where gene conversion could occur by ∑N_ij_(N_ij_-1)/2, where N_ij _is the intact gene numbers in subfamily i within species j, and N must be higher than 2 for gene conversion to be detected by the GeneConv program. The chi test was used to detect statistical significance in the difference in number of gene conversion events occurring between high cysteine and high glycine/tyrosine KRTAP. The Pearson correlation coefficient of the number of genes in each subfamily was determined for pairs of species (table 1). The evolutionary dynamics of the high cysteine and high glycine/tyrosine KRTAP genes was evaluated by comparing the values of the Pearson correlation coefficients.

## Authors' contributions

DDW, DMI and YPZ designed the research and outlined the manuscript together, and DDW drafted the manuscript. ALL authors have read and approved the final manuscript.

## Supplementary Material

Additional file 1 table 1. KRTAP genes in the human, chimpanzee, rhesus macaque, dog, mouse, rat, opossum, and platypus genomes. Click here for file

Additional file 2 figure 1–figure 17. Figure 1–Figure 16 are the phylogenetic trees of high/ultrahigh cysteine KAPs of human (Figure 1), chimpanzee (Figure 2), rhesus macaque(figure 3), dog (figure 4), mouse (figure 5), rat (figure 6), opossum (figure 7), platypus(figure 8), human and chimpanzee (figure 9), human and rhesus macaque (figure 10), human and dog (figure 11), human and mouse (figure 12), human and rat (figure 13), human and opossum (figure 14), human and platypus (figure 15), mouse and rat (figure 16). Figure 17 is the phylogenetic tree of high glycine/tyrosine KRTAPs. h represents human, c is chimpanzee, rh is rhesus macaque, d-dog, m-mouse, r is rat, o is opossum, and p is platypus. The values on the branches are reliabilities, which are evaluated by the interior branch tests with 1,000 replications. Only values higher than 50% are noted. Click here for file

Additional file 3 table 2. Pairs of genes with significant statistical support for gene conversion. The significances are calculated by Bonferroni-corrected method. Click here for file

Additional file 4 table 3. Results of recombination detection by RDP2 program with algorithms: RDP, BootScan, MaxChi and Chimaera with 1,000 permutations. Sequences were considered linear. The highest acceptable P value cut-off was set to 0.01, and the Bonferroni correction was employed. The numbers are the unique events (recombination signals). Click here for file

Additional file 5 human HS-KRTAP aligned protein sequences. The KRTAP5-12 protein sequence was not used for alignment as it is too short. Click here for file

Additional file 6 Aligned human HGT-KRTAP protein sequences. Click here for file
