# Correction: Molecular evolution of the keratin associated protein gene family in mammals, role in the evolution of mammalian hair

**DOI:** 10.1186/1471-2148-9-213

**Published:** 2009-08-25

**Authors:** Dong-Dong Wu, David M Irwin, Ya-Ping Zhang

**Affiliations:** 1 State Key Laboratory of Genetic Resources and Evolution, Kunming Institute of Zoology, Chinese Academy of Sciences, Kunming, PR China; 2 Laboratory for Conservation and Utilization of Bio-resource, Yunnan University, Kunming 650091, PR China; 3 Graduate School of the Chinese Academy of Sciences, Beijing, PR China; 4 Department of Laboratory Medicine and Pathobiology and the Banting and Best Diabetes Centre, University of Toronto, Ontario, Canada

## Abstract

Correction to Wu DD, Irwin DM, Zhang YP: **Molecular evolution of the keratin associated protein gene family in mammals, role in the evolution of mammalian hair**. *BMC Evol Biol *2008, 8:241.

## Correction

In the publication of our work [1], in the table two: Amino acid composition of KRTAPs subfamily genes in mammals, the subfamily 30 should be deleted, and the subfamily 34 should be corrected as 30, 35 should be corrected as 34, and 36 should be corrected as 35. Here, we provide a table 1, which is a corrected version of table two. In the additional file 1, mouse gene repertoire in the table S1, at the line 42, the KRTAP34p1 should be corrected as KRTAP30p1; from the line 177 to 189, the chromosomes should be chr1. We provide a correction of additional file 1. We regret any inconvenience caused to researchers.

**Table 1:** Amino acid composition of KRTAPs subfamily genes in mammals.

HS-KRTAP	subfamily	C	G	L	P	Q	S	T	Y
	1	26.57	10.04	1.67	9.22	6.84	15.55	8.04	1.88
	2	29.1	4.42	1.44	14.4	6.03	10.33	9.25	0.56
	3	19.93	4.72	8.01	15.6	2.9	8.53	10.84	1.53
	4	37.37	2.98	1	10.57	5.47	16.21	7.91	0.51
	5	35.86	23.67	0.1	5.01	3.39	19.25	0.65	0.27
	9	35.26	2.65	1.02	11.04	7.17	12.74	13.95	1.32
	10	27.61	2.55	3.34	13	6.1	18.76	4.62	0.52
	11	13.1	8.08	4.08	8.24	7.45	14.75	12.16	2.43
	12	22.86	2.57	2.17	13.81	6.18	21.12	4.02	1.33
	13	11.47	10.61	5.73	7.41	4.17	21.31	5.88	7.49
	17	36.06	31.44	0.26	4.49	4.23	9.91	3.17	0
	24	9.77	5.05	7.45	9.38	3.99	17.55	7.31	7.18
	25	7	5.07	5.56	8.21	6.28	19.08	3.86	6.28
	26	11.3	9.04	8.47	11.45	4.01	18.08	5.14	3.24
	27	8.71	4.07	6.59	7.81	7.81	18.06	7.73	1.38
	28	39.54	33.66	0.01	1.6	3.93	5.8	2.69	1.02
	29	16.27	6.01	3.34	11.42	8.33	16.7	7.84	2.39
	30	50.73	4.65	0.17	9.54	11.11	4.42	8.55	0.23
	31	26.53	1.02	3.06	11.9	4.76	15.08	10.66	0.11
	32	38.72	3.4	1.91	16.17	4.26	10	8.94	0.21
	33	32.18	5.42	2.65	15.29	1.52	9.17	3.19	0.11
	34	9.19	8.38	4.32	11.08	4.05	21.62	8.11	5.68
	35	10.34	4.6	8.05	11.49	6.9	20.69	6.9	1.15

HGT-KRTAP	6	13.61	40.26	4.87	0.19	0.05	7.59	0.29	22.87
	7	8.81	19.16	4.79	7.28	0.19	11.49	6.13	12.26
	8	5.72	23.97	3.94	7.16	0	8.59	2.15	20.04
	19	6.07	36.52	4.22	1.38	0.33	10.8	0.33	19.96
	20	13.9	37.61	4.61	1.73	0.26	5.72	0.23	24.2
	21	17.2	36.82	0.93	1.03	0.24	12.6	0.87	20.98

The average amino acid content (%) of high cysteine (HS) and high glycine/tyrosine (HGT) KRTAP subfamily genes is shown. C (cysteine), G (glycine), L (leucine), P (proine), Q (glutamine), S (serine), T (threonine) and Y (tyrosine) are single letter codes for amino acids that are abundant in some KRTAP proteins.

## Supplementary Material

Additional file 1 **Table S1 – KRTAP genes in the human, chimpanzee, rhesus macaque, dog, mouse, rat, opossum, and platypus genomes.** Click here for file
