# Supplementary material for: Molecular evolution of the keratin associated protein gene family in mammals, role in the evolution of mammalian hair
Source: BMC Evol Biol. 2008 Aug 23;8:241. doi: 10.1186/1471-2148-8-241 (PMC2528016; doi:10.1186/1471-2148-8-241)

Figure1

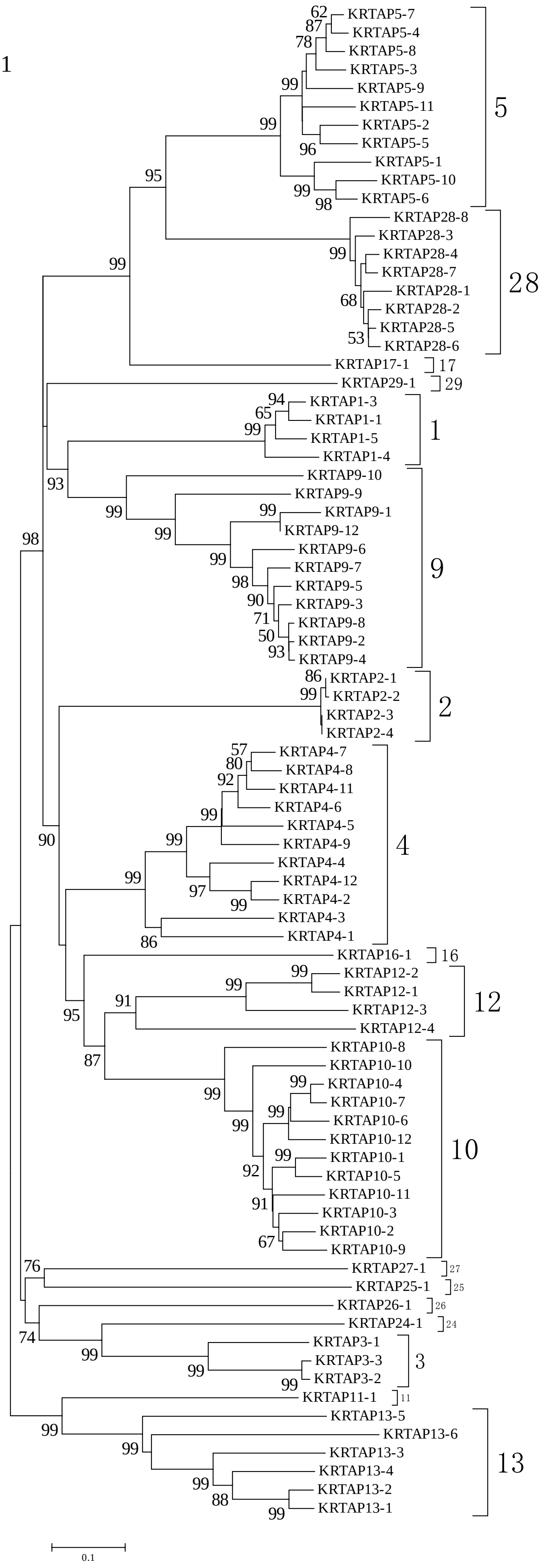
KRTAP5-7
KRTAP5-4
KRTAP5-8
KRTAP5-3
KRTAP5-9
KRTAP5-11
KRTAP5-2
KRTAP5-5
KRTAP5-1
KRTAP5-10
KRTAP5-6
5
KRTAP28-8
KRTAP28-3
KRTAP28-4
KRTAP28-7
KRTAP28-1
KRTAP28-2
KRTAP28-5
KRTAP28-6
28
KRTAP17-1
17
KRTAP29-1
29
KRTAP1-3
KRTAP1-1
KRTAP1-5
KRTAP1-4
1
KRTAP9-10
KRTAP9-9
KRTAP9-1
KRTAP9-12
KRTAP9-6
KRTAP9-7
KRTAP9-5
KRTAP9-3
KRTAP9-8
KRTAP9-2
KRTAP9-4
9
KRTAP2-1
KRTAP2-2
KRTAP2-3
KRTAP2-4
2
KRTAP4-7
KRTAP4-8
KRTAP4-11
KRTAP4-6
KRTAP4-5
KRTAP4-9
KRTAP4-4
KRTAP4-12
KRTAP4-2
KRTAP4-3
KRTAP4-1
4
KRTAP16-1
16
KRTAP12-2
KRTAP12-1
KRTAP12-3
KRTAP12-4
12
KRTAP10-8
KRTAP10-10
KRTAP10-4
KRTAP10-7
KRTAP10-6
KRTAP10-12
KRTAP10-1
KRTAP10-5
KRTAP10-11
KRTAP10-3
KRTAP10-2
KRTAP10-9
10
KRTAP27-1
27
KRTAP25-1
25
KRTAP26-1
26
KRTAP24-1
24
KRTAP3-1
KRTAP3-3
KRTAP3-2
3
KRTAP11-1
11
KRTAP13-5
KRTAP13-6
KRTAP13-3
KRTAP13-4
KRTAP13-2
KRTAP13-1
13
0.1

Figure2

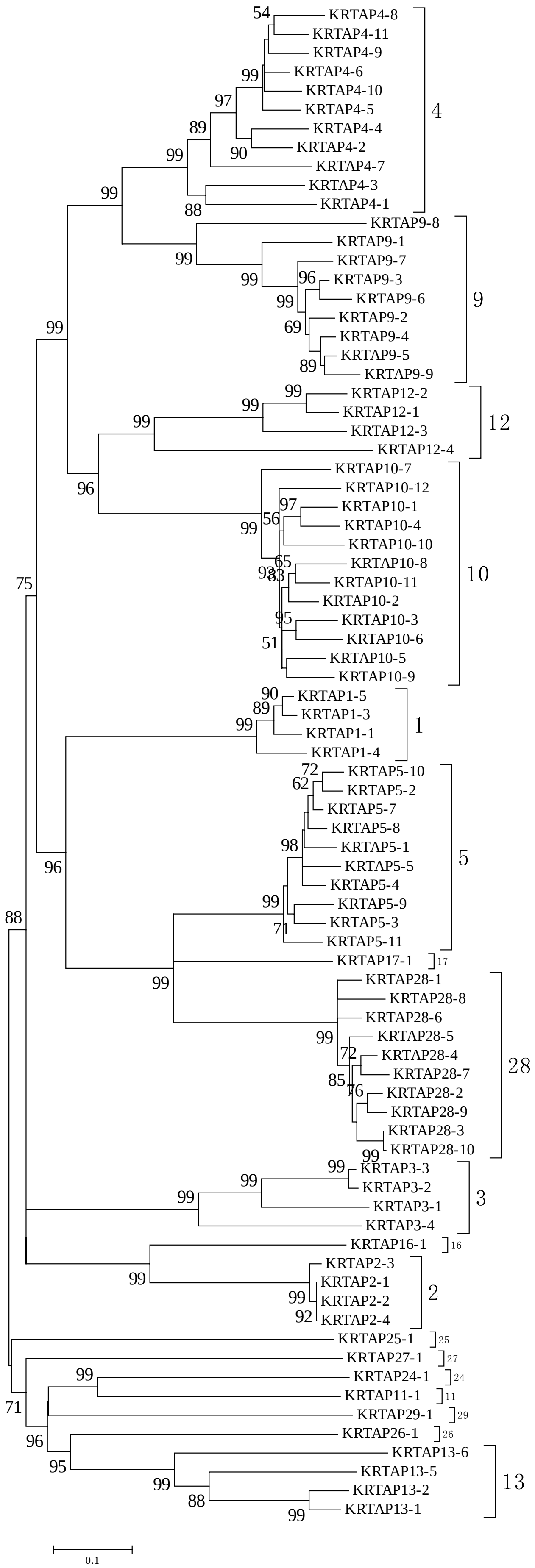
KRTAP4-8
KRTAP4-11
KRTAP4-9
KRTAP4-6
KRTAP4-10
KRTAP4-5
KRTAP4-4
KRTAP4-2
KRTAP4-7
KRTAP4-3
KRTAP4-1
4
KRTAP9-8
KRTAP9-1
KRTAP9-7
KRTAP9-3
KRTAP9-6
KRTAP9-2
KRTAP9-4
KRTAP9-5
KRTAP9-9
9
KRTAP12-2
KRTAP12-1
KRTAP12-3
KRTAP12-4
12
KRTAP10-7
KRTAP10-12
KRTAP10-1
KRTAP10-4
KRTAP10-10
KRTAP10-8
KRTAP10-11
KRTAP10-2
KRTAP10-3
KRTAP10-6
KRTAP10-5
KRTAP10-9
10
KRTAP1-5
KRTAP1-3
KRTAP1-1
KRTAP1-4
1
KRTAP5-10
KRTAP5-2
KRTAP5-7
KRTAP5-8
KRTAP5-1
KRTAP5-5
KRTAP5-4
KRTAP5-9
KRTAP5-3
KRTAP5-11
5
KRTAP17-1
17
KRTAP28-1
KRTAP28-8
KRTAP28-6
KRTAP28-5
KRTAP28-4
KRTAP28-7
KRTAP28-2
KRTAP28-9
KRTAP28-3
KRTAP28-10
28
KRTAP3-3
KRTAP3-2
KRTAP3-1
KRTAP3-4
3
KRTAP16-1
16
KRTAP2-3
KRTAP2-1
KRTAP2-2
KRTAP2-4
2
KRTAP25-1
25
KRTAP27-1
27
KRTAP24-1
24
KRTAP11-1
11
KRTAP29-1
29
KRTAP26-1
26
KRTAP13-6
KRTAP13-5
KRTAP13-2
KRTAP13-1
13
0.1

Figure3

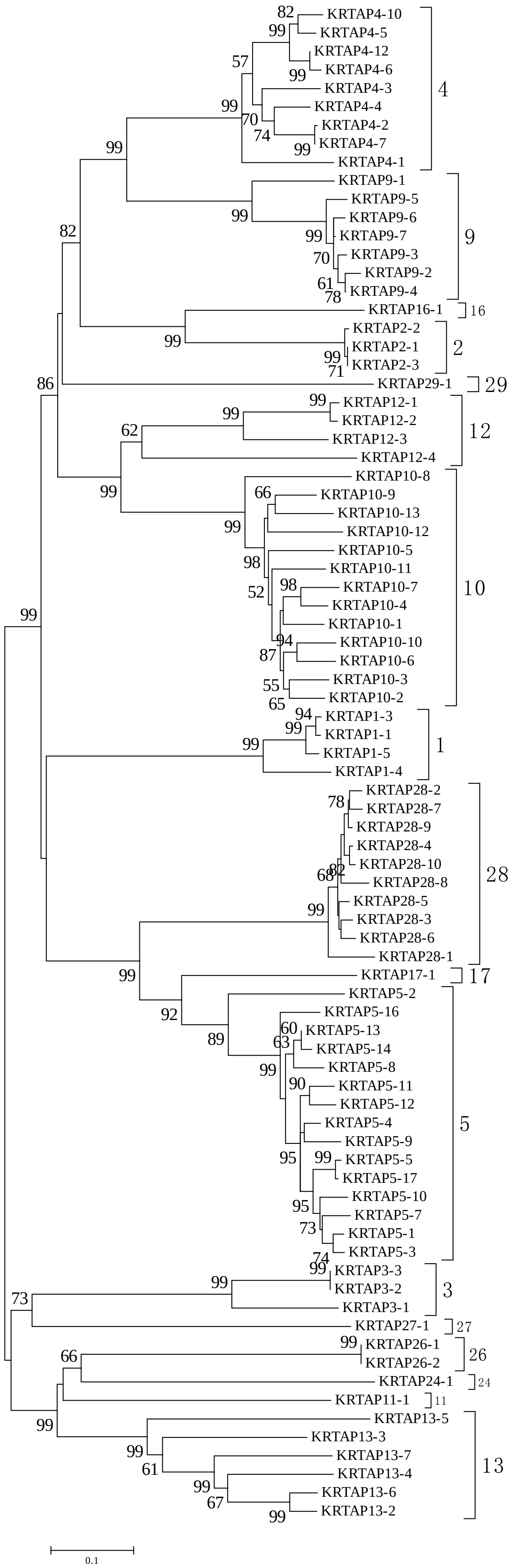

KRTAP4-10
KRTAP4-5
KRTAP4-12
KRTAP4-6
KRTAP4-3
KRTAP4-4
KRTAP4-2
KRTAP4-7
KRTAP4-1
4
KRTAP9-1
KRTAP9-5
KRTAP9-6
KRTAP9-7
KRTAP9-3
KRTAP9-2
KRTAP9-4
9
KRTAP16-1
16
KRTAP2-2
KRTAP2-1
KRTAP2-3
2
KRTAP29-1
29
KRTAP12-1
KRTAP12-2
KRTAP12-3
KRTAP12-4
12
KRTAP10-8
KRTAP10-9
KRTAP10-13
KRTAP10-12
KRTAP10-5
KRTAP10-11
KRTAP10-7
KRTAP10-4
KRTAP10-1
KRTAP10-10
KRTAP10-6
KRTAP10-3
KRTAP10-2
10
KRTAP1-3
KRTAP1-1
KRTAP1-5
KRTAP1-4
1
KRTAP28-2
KRTAP28-7
KRTAP28-9
KRTAP28-4
KRTAP28-10
KRTAP28-8
KRTAP28-5
KRTAP28-3
KRTAP28-6
KRTAP28-1
28
KRTAP17-1
17
KRTAP5-2
KRTAP5-16
KRTAP5-13
KRTAP5-14
KRTAP5-8
KRTAP5-11
KRTAP5-12
KRTAP5-4
KRTAP5-9
KRTAP5-5
KRTAP5-17
KRTAP5-10
KRTAP5-7
KRTAP5-1
KRTAP5-3
5
KRTAP3-3
KRTAP3-2
KRTAP3-1
3
KRTAP27-1
27
KRTAP26-1
KRTAP26-2
26
KRTAP24-1
24
KRTAP11-1
11
KRTAP13-5
KRTAP13-3
KRTAP13-7
KRTAP13-4
KRTAP13-6
KRTAP13-2
13
0.1

Figure4

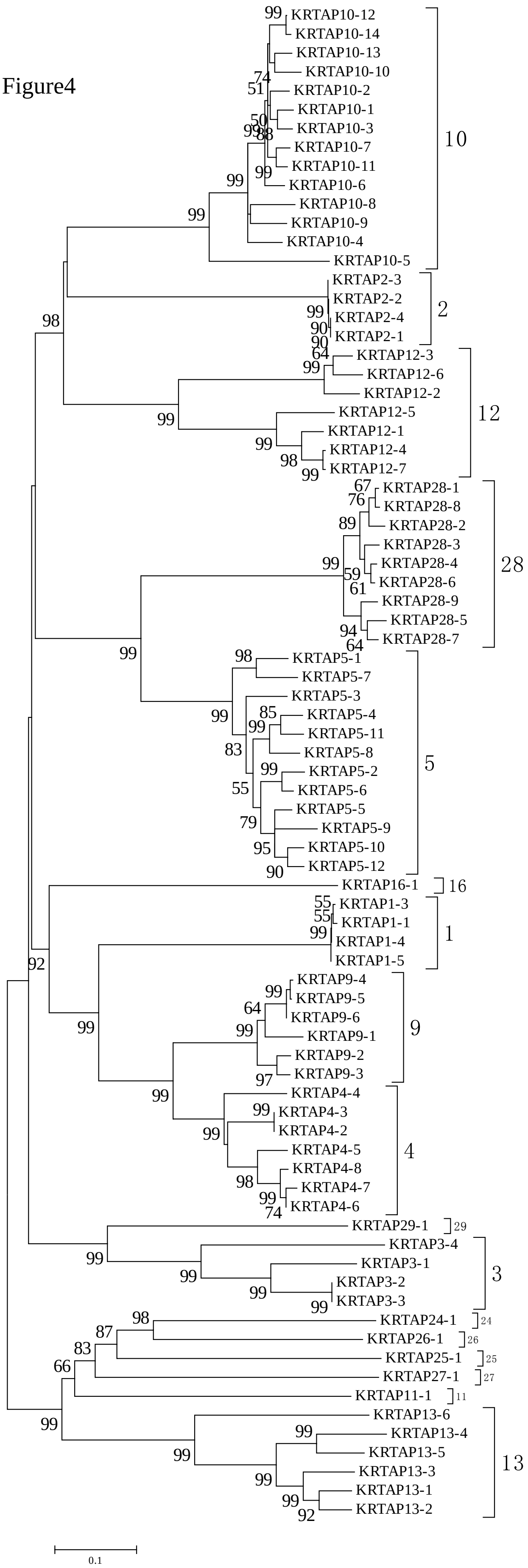
KRTAP10-12
KRTAP10-14
KRTAP10-13
KRTAP10-10
KRTAP10-2
KRTAP10-1
KRTAP10-3
KRTAP10-7
KRTAP10-11
KRTAP10-6
KRTAP10-8
KRTAP10-9
KRTAP10-4
KRTAP10-5
10
KRTAP2-3
KRTAP2-2
KRTAP2-4
KRTAP2-1
2
KRTAP12-3
KRTAP12-6
KRTAP12-2
KRTAP12-5
KRTAP12-1
KRTAP12-4
KRTAP12-7
12
KRTAP28-1
KRTAP28-8
KRTAP28-2
KRTAP28-3
KRTAP28-4
KRTAP28-6
KRTAP28-9
KRTAP28-5
KRTAP28-7
28
KRTAP5-1
KRTAP5-7
KRTAP5-3
KRTAP5-4
KRTAP5-11
KRTAP5-8
KRTAP5-2
KRTAP5-6
KRTAP5-5
KRTAP5-9
KRTAP5-10
KRTAP5-12
5
KRTAP16-1
16
KRTAP1-3
KRTAP1-1
KRTAP1-4
KRTAP1-5
1
KRTAP9-4
KRTAP9-5
KRTAP9-6
KRTAP9-1
KRTAP9-2
KRTAP9-3
9
KRTAP4-4
KRTAP4-3
KRTAP4-2
KRTAP4-5
KRTAP4-8
KRTAP4-7
KRTAP4-6
4
KRTAP29-1
29
KRTAP3-4
KRTAP3-1
KRTAP3-2
KRTAP3-3
3
KRTAP24-1
24
KRTAP26-1
26
KRTAP25-1
25
KRTAP27-1
27
KRTAP11-1
11
KRTAP13-6
KRTAP13-4
KRTAP13-5
KRTAP13-3
KRTAP13-1
KRTAP13-2
13
0.1

Figure5

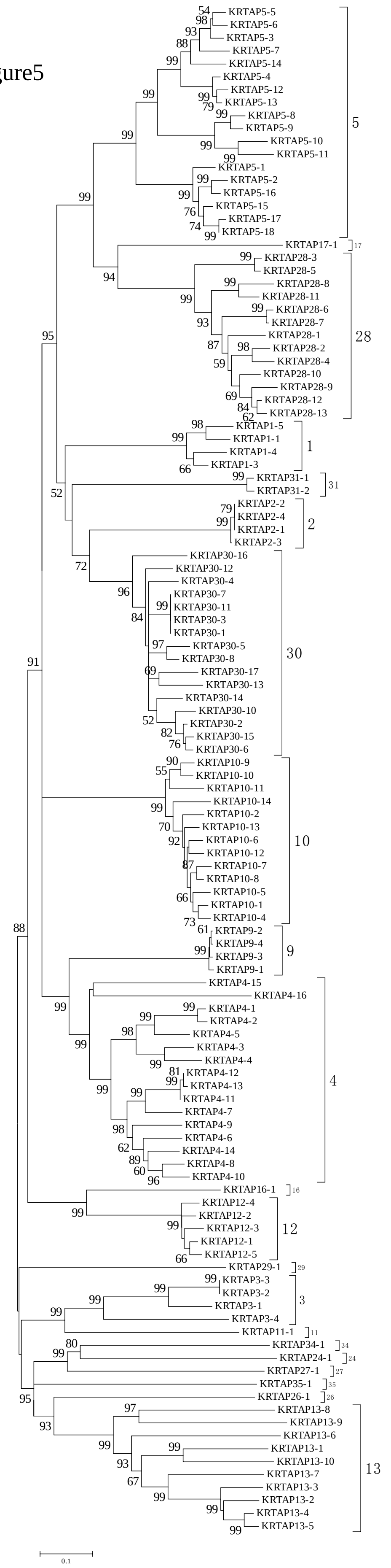
KRTAP5-5
KRTAP5-6
KRTAP5-3
KRTAP5-7
KRTAP5-14
KRTAP5-4
KRTAP5-12
KRTAP5-13
KRTAP5-8
KRTAP5-9
KRTAP5-10
KRTAP5-11
KRTAP5-1
KRTAP5-2
KRTAP5-16
KRTAP5-15
KRTAP5-17
KRTAP5-18
5
KRTAP17-1
17
KRTAP28-3
KRTAP28-5
KRTAP28-8
KRTAP28-11
KRTAP28-6
KRTAP28-7
KRTAP28-1
KRTAP28-2
KRTAP28-4
KRTAP28-10
KRTAP28-9
KRTAP28-12
KRTAP28-13
28
KRTAP1-5
KRTAP1-1
KRTAP1-4
KRTAP1-3
1
KRTAP31-1
KRTAP31-2
31
KRTAP2-2
KRTAP2-4
KRTAP2-1
KRTAP2-3
2
KRTAP30-16
KRTAP30-12
KRTAP30-4
KRTAP30-7
KRTAP30-11
KRTAP30-3
KRTAP30-1
KRTAP30-5
KRTAP30-8
KRTAP30-17
KRTAP30-13
KRTAP30-14
KRTAP30-10
KRTAP30-2
KRTAP30-15
KRTAP30-6
30
KRTAP10-9
KRTAP10-10
KRTAP10-11
KRTAP10-14
KRTAP10-2
KRTAP10-13
KRTAP10-6
KRTAP10-12
KRTAP10-7
KRTAP10-8
KRTAP10-5
KRTAP10-1
KRTAP10-4
10
KRTAP9-2
KRTAP9-4
KRTAP9-3
KRTAP9-1
9
KRTAP4-15
KRTAP4-16
KRTAP4-1
KRTAP4-2
KRTAP4-5
KRTAP4-3
KRTAP4-4
KRTAP4-12
KRTAP4-13
KRTAP4-11
KRTAP4-7
KRTAP4-9
KRTAP4-6
KRTAP4-14
KRTAP4-8
KRTAP4-10
4
KRTAP16-1
16
KRTAP12-4
KRTAP12-2
KRTAP12-3
KRTAP12-1
KRTAP12-5
12
KRTAP29-1
29
KRTAP3-3
KRTAP3-2
KRTAP3-1
KRTAP3-4
3
KRTAP11-1
11
KRTAP34-1
34
KRTAP24-1
24
KRTAP27-1
27
KRTAP35-1
35
KRTAP26-1
26
KRTAP13-8
KRTAP13-9
KRTAP13-6
KRTAP13-1
KRTAP13-10
KRTAP13-7
KRTAP13-3
KRTAP13-2
KRTAP13-4
KRTAP13-5
13
0.1

Figure6

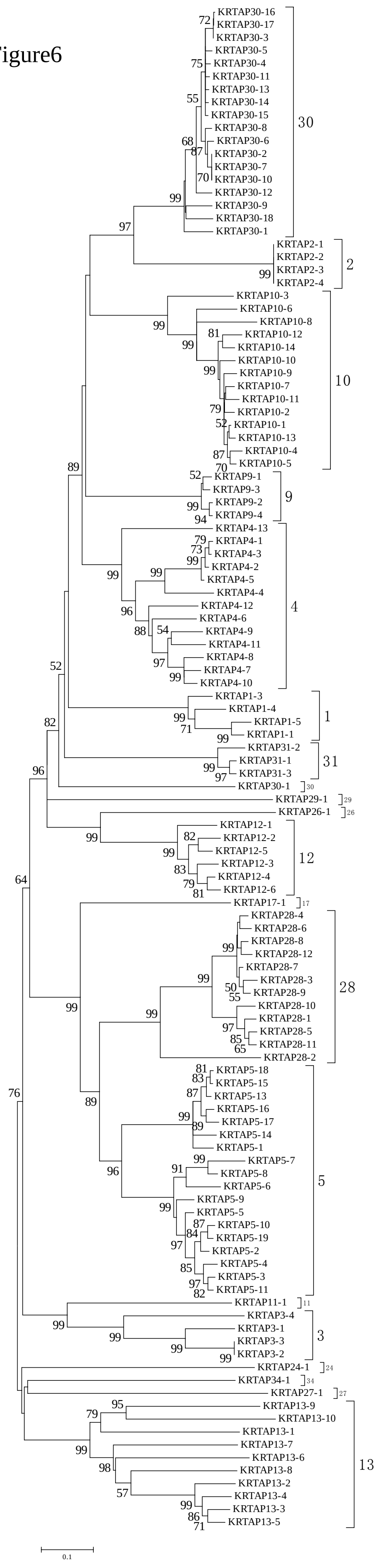

KRTAP30-16
KRTAP30-17
KRTAP30-3
KRTAP30-5
KRTAP30-4
KRTAP30-11
KRTAP30-13
KRTAP30-14
KRTAP30-15
KRTAP30-8
KRTAP30-6
KRTAP30-2
KRTAP30-7
KRTAP30-10
KRTAP30-12
KRTAP30-9
KRTAP30-18
KRTAP30-1
30
KRTAP2-1
KRTAP2-2
KRTAP2-3
KRTAP2-4
2
KRTAP10-3
KRTAP10-6
KRTAP10-8
KRTAP10-12
KRTAP10-14
KRTAP10-10
KRTAP10-9
KRTAP10-7
KRTAP10-11
KRTAP10-2
KRTAP10-1
KRTAP10-13
KRTAP10-4
KRTAP10-5
10
KRTAP9-1
KRTAP9-3
KRTAP9-2
KRTAP9-4
9
KRTAP4-13
KRTAP4-1
KRTAP4-3
KRTAP4-2
KRTAP4-5
KRTAP4-4
KRTAP4-12
KRTAP4-6
KRTAP4-9
KRTAP4-11
KRTAP4-8
KRTAP4-7
KRTAP4-10
4
KRTAP1-3
KRTAP1-4
KRTAP1-5
KRTAP1-1
1
KRTAP31-2
KRTAP31-1
KRTAP31-3
31
KRTAP30-1
30
KRTAP29-1
29
KRTAP26-1
26
KRTAP12-1
KRTAP12-2
KRTAP12-5
KRTAP12-3
KRTAP12-4
KRTAP12-6
12
KRTAP17-1
17
KRTAP28-4
KRTAP28-6
KRTAP28-8
KRTAP28-12
KRTAP28-7
KRTAP28-3
KRTAP28-9
KRTAP28-10
KRTAP28-1
KRTAP28-5
KRTAP28-11
KRTAP28-2
28
KRTAP5-18
KRTAP5-15
KRTAP5-13
KRTAP5-16
KRTAP5-17
KRTAP5-14
KRTAP5-1
KRTAP5-7
KRTAP5-8
KRTAP5-6
KRTAP5-9
KRTAP5-5
KRTAP5-10
KRTAP5-19
KRTAP5-2
KRTAP5-4
KRTAP5-3
KRTAP5-11
5
KRTAP11-1
11
KRTAP3-4
KRTAP3-1
KRTAP3-3
KRTAP3-2
3
KRTAP24-1
24
KRTAP34-1
34
KRTAP27-1
27
KRTAP13-9
KRTAP13-10
KRTAP13-1
KRTAP13-7
KRTAP13-6
KRTAP13-8
KRTAP13-2
KRTAP13-4
KRTAP13-3
KRTAP13-5
13
0.1

Figure7

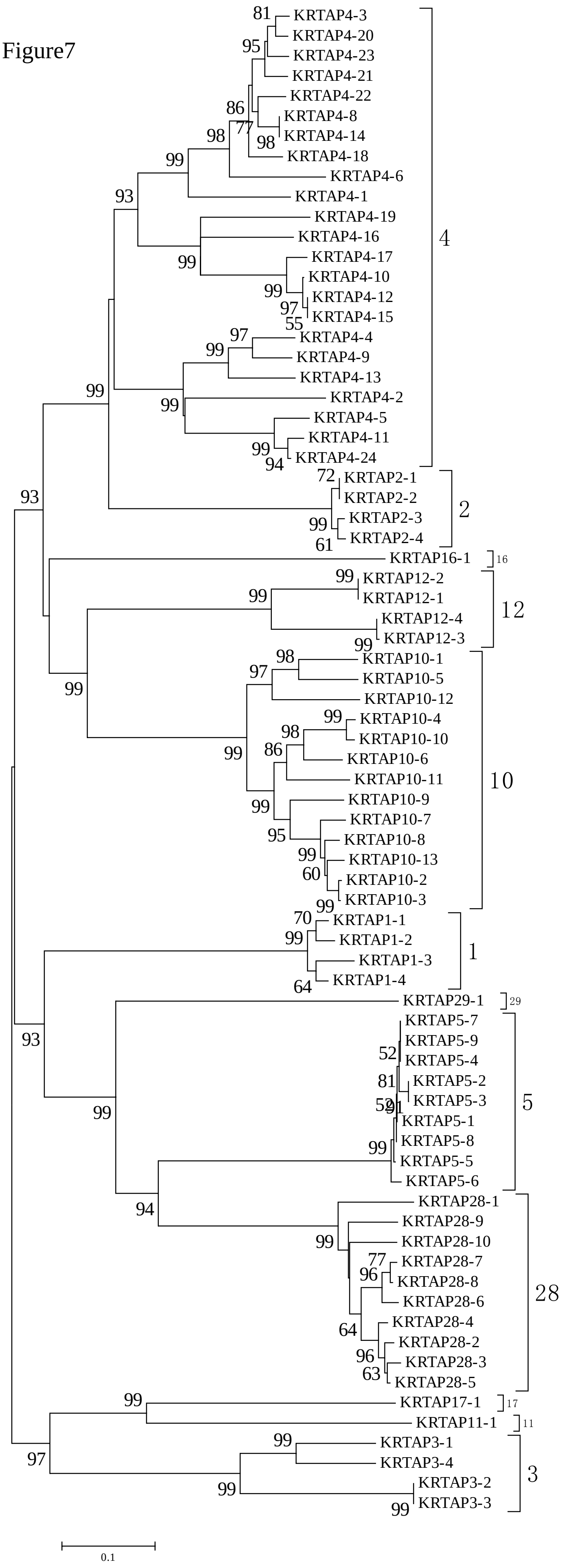
KRTAP4-3
KRTAP4-20
KRTAP4-23
KRTAP4-21
KRTAP4-22
KRTAP4-8
KRTAP4-14
KRTAP4-18
KRTAP4-6
KRTAP4-1
KRTAP4-19
KRTAP4-16
KRTAP4-17
KRTAP4-10
KRTAP4-12
KRTAP4-15
KRTAP4-4
KRTAP4-9
KRTAP4-13
KRTAP4-2
KRTAP4-5
KRTAP4-11
KRTAP4-24
4
KRTAP2-1
KRTAP2-2
KRTAP2-3
KRTAP2-4
2
KRTAP16-1
16
KRTAP12-2
KRTAP12-1
KRTAP12-4
KRTAP12-3
12
KRTAP10-1
KRTAP10-5
KRTAP10-12
KRTAP10-4
KRTAP10-10
KRTAP10-6
KRTAP10-11
KRTAP10-9
KRTAP10-7
KRTAP10-8
KRTAP10-13
KRTAP10-2
KRTAP10-3
10
KRTAP1-1
KRTAP1-2
KRTAP1-3
KRTAP1-4
1
KRTAP29-1
29
KRTAP5-7
KRTAP5-9
KRTAP5-4
KRTAP5-2
KRTAP5-3
KRTAP5-1
KRTAP5-8
KRTAP5-5
KRTAP5-6
5
KRTAP28-1
KRTAP28-9
KRTAP28-10
KRTAP28-7
KRTAP28-8
KRTAP28-6
KRTAP28-4
KRTAP28-2
KRTAP28-3
KRTAP28-5
28
KRTAP17-1
17
KRTAP11-1
11
KRTAP3-1
KRTAP3-4
KRTAP3-2
KRTAP3-3
3
0.1

Figure8

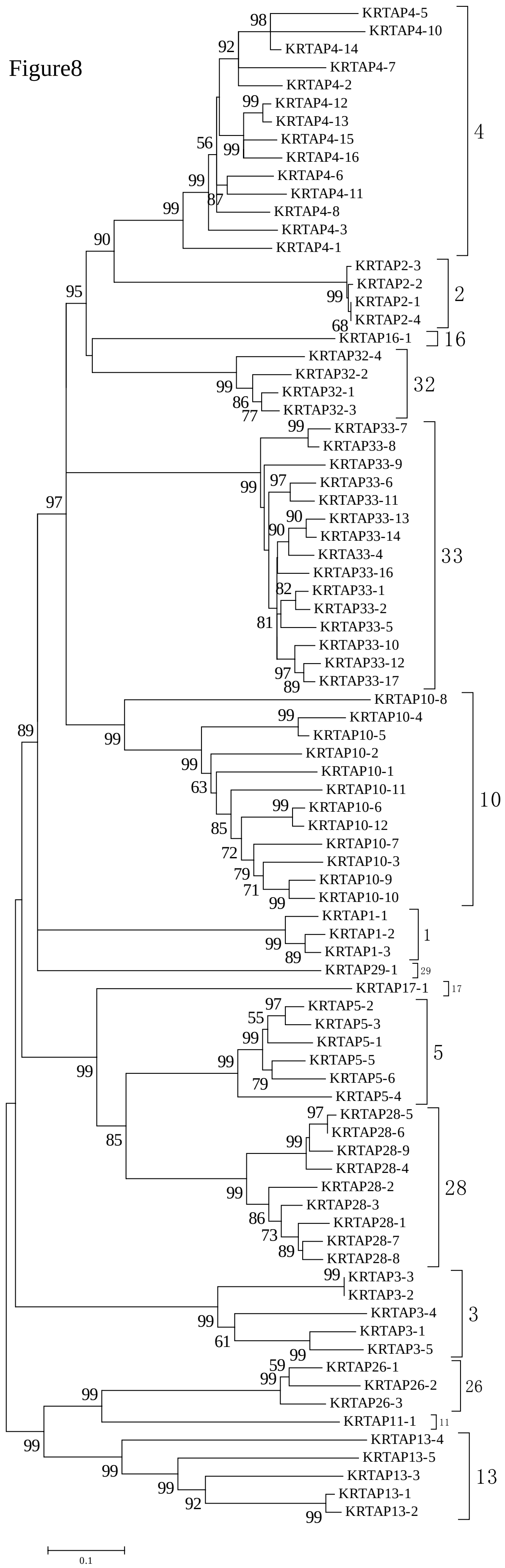
KRTAP4-5
KRTAP4-10
KRTAP4-14
KRTAP4-7
KRTAP4-2
KRTAP4-12
KRTAP4-13
KRTAP4-15
KRTAP4-16
KRTAP4-6
KRTAP4-11
KRTAP4-8
KRTAP4-3
KRTAP4-1
4
KRTAP2-3
KRTAP2-2
KRTAP2-1
KRTAP2-4
2
KRTAP16-1
16
KRTAP32-4
KRTAP32-2
KRTAP32-1
KRTAP32-3
32
KRTAP33-7
KRTAP33-8
KRTAP33-9
KRTAP33-6
KRTAP33-11
KRTAP33-13
KRTAP33-14
KRTA33-4
KRTAP33-16
KRTAP33-1
KRTAP33-2
KRTAP33-5
KRTAP33-10
KRTAP33-12
KRTAP33-17
33
KRTAP10-8
KRTAP10-4
KRTAP10-5
KRTAP10-2
KRTAP10-1
KRTAP10-11
KRTAP10-6
KRTAP10-12
KRTAP10-7
KRTAP10-3
KRTAP10-9
KRTAP10-10
10
KRTAP1-1
KRTAP1-2
KRTAP1-3
1
KRTAP29-1
29
KRTAP17-1
17
KRTAP5-2
KRTAP5-3
KRTAP5-1
KRTAP5-5
KRTAP5-6
KRTAP5-4
5
KRTAP28-5
KRTAP28-6
KRTAP28-9
KRTAP28-4
KRTAP28-2
KRTAP28-3
KRTAP28-1
KRTAP28-7
KRTAP28-8
28
KRTAP3-3
KRTAP3-2
KRTAP3-4
KRTAP3-1
KRTAP3-5
3
KRTAP26-1
KRTAP26-2
KRTAP26-3
26
KRTAP11-1
11
KRTAP13-4
KRTAP13-5
KRTAP13-3
KRTAP13-1
KRTAP13-2
13
0.1

Figure9

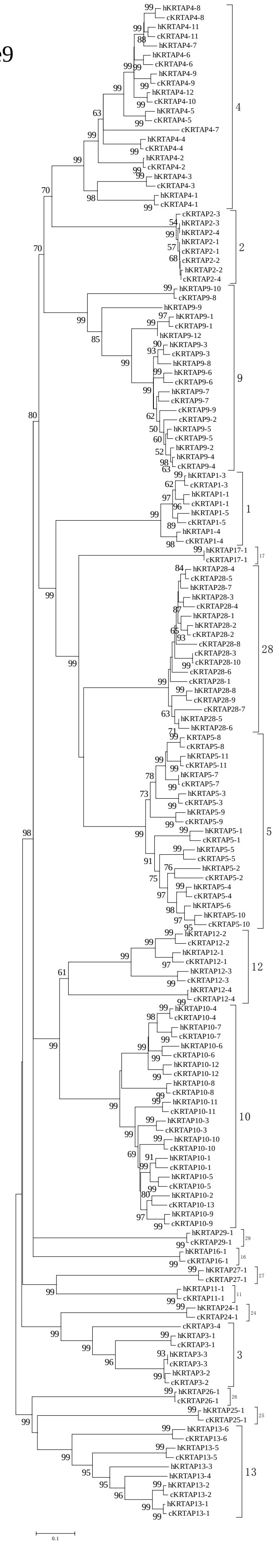
hKRTAP4-8
cKRTAP4-8
hKRTAP4-11
cKRTAP4-11
hKRTAP4-7
hKRTAP4-6
cKRTAP4-6
hKRTAP4-9
cKRTAP4-9
hKRTAP4-12
cKRTAP4-10
hKRTAP4-5
cKRTAP4-5
cKRTAP4-7
hKRTAP4-4
cKRTAP4-4
hKRTAP4-2
cKRTAP4-2
hKRTAP4-3
cKRTAP4-3
hKRTAP4-1
cKRTAP4-1
4
cKRTAP2-3
hKRTAP2-3
hKRTAP2-4
hKRTAP2-1
cKRTAP2-1
cKRTAP2-2
hKRTAP2-2
cKRTAP2-4
2
hKRTAP9-10
cKRTAP9-8
hKRTAP9-9
hKRTAP9-1
cKRTAP9-1
hKRTAP9-12
hKRTAP9-3
cKRTAP9-3
hKRTAP9-8
hKRTAP9-6
cKRTAP9-6
hKRTAP9-7
cKRTAP9-7
cKRTAP9-9
cKRTAP9-2
hKRTAP9-5
cKRTAP9-5
hKRTAP9-2
hKRTAP9-4
cKRTAP9-4
9
hKRTAP1-3
cKRTAP1-3
hKRTAP1-1
cKRTAP1-1
hKRTAP1-5
cKRTAP1-5
hKRTAP1-4
cKRTAP1-4
1
hKRTAP17-1
cKRTAP17-1
17
hKRTAP28-4
cKRTAP28-5
hKRTAP28-7
hKRTAP28-3
cKRTAP28-4
hKRTAP28-1
hKRTAP28-2
cKRTAP28-2
cKRTAP28-8
cKRTAP28-3
cKRTAP28-10
cKRTAP28-6
cKRTAP28-1
hKRTAP28-8
cKRTAP28-9
cKRTAP28-7
hKRTAP28-5
hKRTAP28-6
28
KRTAP5-8
cKRTAP5-8
hKRTAP5-11
cKRTAP5-11
hKRTAP5-7
cKRTAP5-7
hKRTAP5-3
cKRTAP5-3
hKRTAP5-9
cKRTAP5-9
hKRTAP5-1
cKRTAP5-1
hKRTAP5-5
cKRTAP5-5
hKRTAP5-2
cKRTAP5-2
hKRTAP5-4
cKRTAP5-4
hKRTAP5-6
hKRTAP5-10
cKRTAP5-10
5
hKRTAP12-2
cKRTAP12-2
hKRTAP12-1
cKRTAP12-1
hKRTAP12-3
cKRTAP12-3
hKRTAP12-4
cKRTAP12-4
12
hKRTAP10-4
cKRTAP10-4
hKRTAP10-7
cKRTAP10-7
hKRTAP10-6
cKRTAP10-6
hKRTAP10-12
cKRTAP10-12
hKRTAP10-8
cKRTAP10-8
hKRTAP10-11
cKRTAP10-11
hKRTAP10-3
cKRTAP10-3
hKRTAP10-10
cKRTAP10-10
hKRTAP10-1
cKRTAP10-1
hKRTAP10-5
cKRTAP10-5
hKRTAP10-2
cKRTAP10-13
hKRTAP10-9
cKRTAP10-9
10
hKRTAP29-1
cKRTAP29-1
29
hKRTAP16-1
cKRTAP16-1
16
hKRTAP27-1
cKRTAP27-1
27
hKRTAP11-1
cKRTAP11-1
11
hKRTAP24-1
cKRTAP24-1
24
cKRTAP3-4
hKRTAP3-1
cKRTAP3-1
hKRTAP3-3
cKRTAP3-3
hKRTAP3-2
cKRTAP3-2
3
hKRTAP26-1
cKRTAP26-1
26
hKRTAP25-1
cKRTAP25-1
25
hKRTAP13-6
cKRTAP13-6
hKRTAP13-5
cKRTAP13-5
hKRTAP13-3
hKRTAP13-4
hKRTAP13-2
cKRTAP13-2
hKRTAP13-1
cKRTAP13-1
13
0.1

Figure10

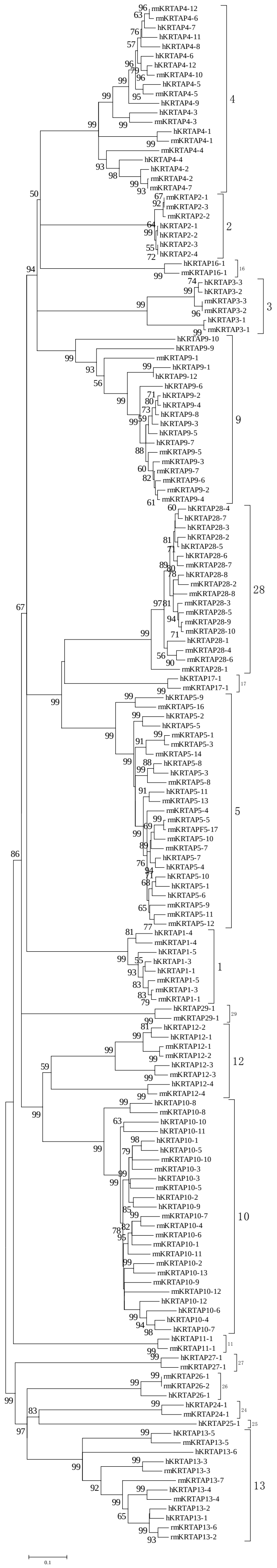

Figure11

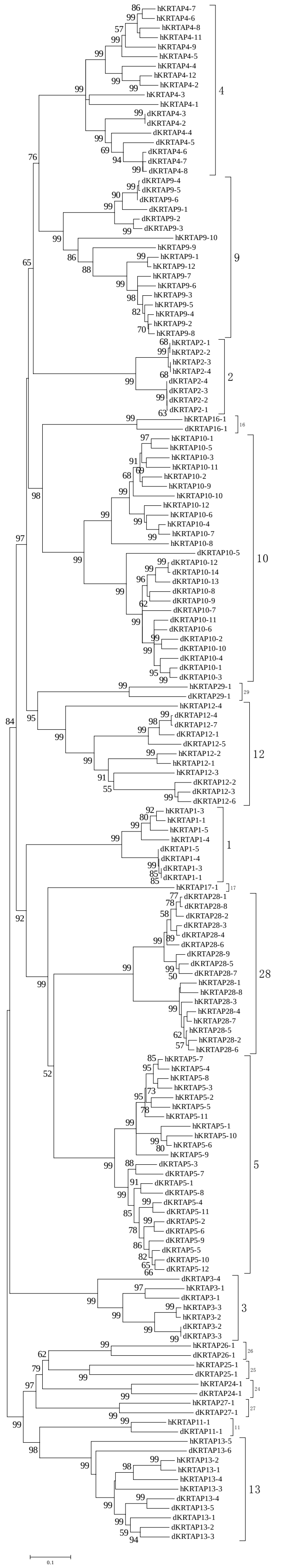
hKRTAP4-7
hKRTAP4-6
hKRTAP4-8
hKRTAP4-11
hKRTAP4-9
hKRTAP4-5
hKRTAP4-4
hKRTAP4-12
hKRTAP4-2
hKRTAP4-3
hKRTAP4-1
dKRTAP4-3
dKRTAP4-2
dKRTAP4-4
dKRTAP4-5
dKRTAP4-6
dKRTAP4-7
dKRTAP4-8
4
dKRTAP9-4
dKRTAP9-5
dKRTAP9-6
dKRTAP9-1
dKRTAP9-2
dKRTAP9-3
hKRTAP9-10
hKRTAP9-9
hKRTAP9-1
hKRTAP9-12
hKRTAP9-7
hKRTAP9-6
hKRTAP9-3
hKRTAP9-5
hKRTAP9-4
hKRTAP9-2
hKRTAP9-8
9
hKRTAP2-1
hKRTAP2-2
hKRTAP2-3
hKRTAP2-4
dKRTAP2-4
dKRTAP2-3
dKRTAP2-2
dKRTAP2-1
2
hKRTAP16-1
dKRTAP16-1
16
hKRTAP10-1
hKRTAP10-5
hKRTAP10-3
hKRTAP10-11
hKRTAP10-2
hKRTAP10-9
hKRTAP10-10
hKRTAP10-12
hKRTAP10-6
hKRTAP10-4
hKRTAP10-7
hKRTAP10-8
dKRTAP10-5
dKRTAP10-12
dKRTAP10-14
dKRTAP10-13
dKRTAP10-8
dKRTAP10-9
dKRTAP10-7
dKRTAP10-11
dKRTAP10-6
dKRTAP10-2
dKRTAP10-10
dKRTAP10-4
dKRTAP10-1
dKRTAP10-3
10
hKRTAP29-1
dKRTAP29-1
29
hKRTAP12-4
dKRTAP12-4
dKRTAP12-7
dKRTAP12-1
dKRTAP12-5
hKRTAP12-2
hKRTAP12-1
hKRTAP12-3
dKRTAP12-2
dKRTAP12-3
dKRTAP12-6
12
hKRTAP1-3
hKRTAP1-1
hKRTAP1-5
hKRTAP1-4
dKRTAP1-5
dKRTAP1-4
dKRTAP1-3
dKRTAP1-1
1
hKRTAP17-1
17
dKRTAP28-1
dKRTAP28-8
dKRTAP28-2
dKRTAP28-3
dKRTAP28-4
dKRTAP28-6
dKRTAP28-9
dKRTAP28-5
dKRTAP28-7
hKRTAP28-1
hKRTAP28-8
hKRTAP28-3
hKRTAP28-4
hKRTAP28-7
hKRTAP28-5
hKRTAP28-2
hKRTAP28-6
28
hKRTAP5-7
hKRTAP5-4
hKRTAP5-8
hKRTAP5-3
hKRTAP5-2
hKRTAP5-5
hKRTAP5-11
hKRTAP5-1
hKRTAP5-10
hKRTAP5-6
hKRTAP5-9
dKRTAP5-3
dKRTAP5-7
dKRTAP5-1
dKRTAP5-8
dKRTAP5-4
dKRTAP5-11
dKRTAP5-2
dKRTAP5-6
dKRTAP5-9
dKRTAP5-5
dKRTAP5-10
dKRTAP5-12
5
dKRTAP3-4
hKRTAP3-1
dKRTAP3-1
hKRTAP3-3
hKRTAP3-2
dKRTAP3-2
dKRTAP3-3
3
hKRTAP26-1
dKRTAP26-1
26
hKRTAP25-1
dKRTAP25-1
25
hKRTAP24-1
dKRTAP24-1
24
hKRTAP27-1
dKRTAP27-1
27
hKRTAP11-1
dKRTAP11-1
11
hKRTAP13-5
dKRTAP13-6
hKRTAP13-2
hKRTAP13-1
hKRTAP13-4
hKRTAP13-3
dKRTAP13-4
dKRTAP13-5
dKRTAP13-1
dKRTAP13-2
dKRTAP13-3
13
0.1

Figure12

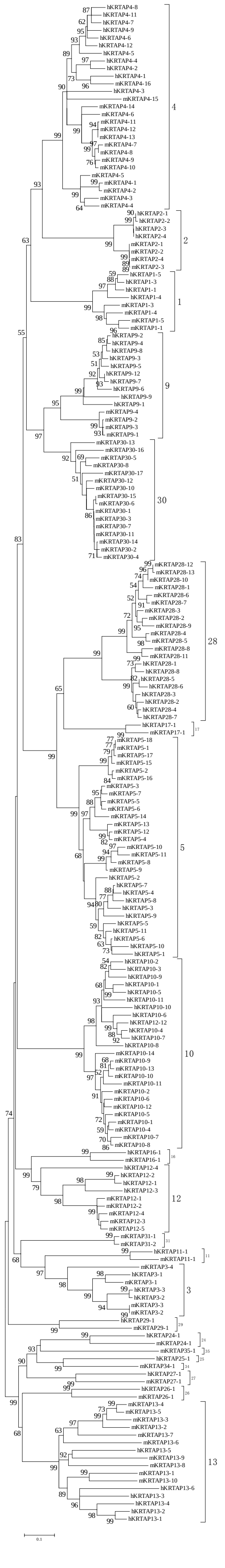

Figure13

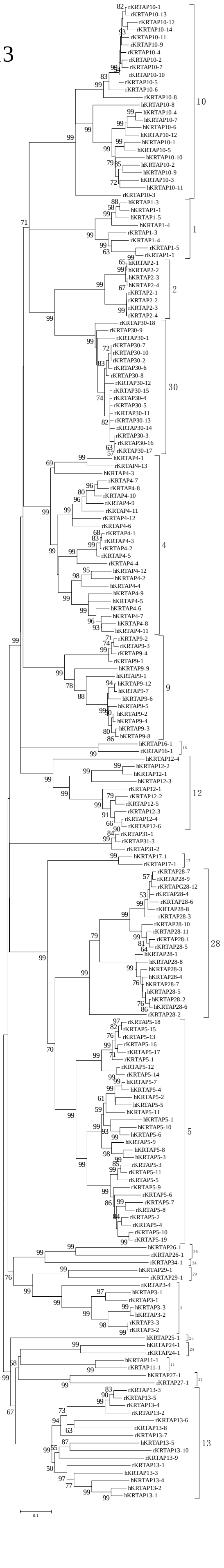
rKRTAP10-1
rKRTAP10-13
rKRTAP10-12
rKRTAP10-14
rKRTAP10-11
rKRTAP10-9
rKRTAP10-4
rKRTAP10-2
rKRTAP10-7
rKRTAP10-10
rKRTAP10-5
rKRTAP10-6
rKRTAP10-8
hKRTAP10-8
hKRTAP10-4
hKRTAP10-7
hKRTAP10-6
hKRTAP10-12
hKRTAP10-1
hKRTAP10-5
hKRTAP10-10
hKRTAP10-2
hKRTAP10-9
hKRTAP10-3
hKRTAP10-11
rKRTAP10-3
10
hKRTAP1-3
hKRTAP1-1
hKRTAP1-5
hKRTAP1-4
rKRTAP1-3
rKRTAP1-4
rKRTAP1-5
rKRTAP1-1
1
hKRTAP2-1
hKRTAP2-2
hKRTAP2-3
hKRTAP2-4
rKRTAP2-1
rKRTAP2-2
rKRTAP2-3
rKRTAP2-4
2
rKRTAP30-18
rKRTAP30-9
rKRTAP30-1
rKRTAP30-7
rKRTAP30-10
rKRTAP30-2
rKRTAP30-6
rKRTAP30-8
rKRTAP30-12
rKRTAP30-15
rKRTAP30-4
rKRTAP30-5
rKRTAP30-11
rKRTAP30-13
rKRTAP30-14
rKRTAP30-3
rKRTAP30-16
rKRTAP30-17
30
hKRTAP4-1
rKRTAP4-13
hKRTAP4-3
rKRTAP4-7
rKRTAP4-8
rKRTAP4-10
rKRTAP4-9
rKRTAP4-11
rKRTAP4-12
rKRTAP4-6
rKRTAP4-1
rKRTAP4-3
rKRTAP4-2
rKRTAP4-5
rKRTAP4-4
hKRTAP4-12
hKRTAP4-2
hKRTAP4-4
hKRTAP4-9
hKRTAP4-5
hKRTAP4-6
hKRTAP4-7
hKRTAP4-8
hKRTAP4-11
4
rKRTAP9-2
rKRTAP9-3
rKRTAP9-4
rKRTAP9-1
hKRTAP9-9
hKRTAP9-1
hKRTAP9-12
hKRTAP9-7
hKRTAP9-6
hKRTAP9-5
hKRTAP9-2
hKRTAP9-4
hKRTAP9-3
hKRTAP9-8
9
hKRTAP16-1
rKRTAP16-1
hKRTAP12-4
hKRTAP12-2
hKRTAP12-1
hKRTAP12-3
rKRTAP12-1
rKRTAP12-2
rKRTAP12-5
rKRTAP12-3
rKRTAP12-4
rKRTAP12-6
12
rKRTAP31-1
rKRTAP31-3
rKRTAP31-2
hKRTAP17-1
rKRTAP17-1
rKRTAP28-7
rKRTAP28-9
rKRTAPG28-12
rKRTAP28-4
rKRTAP28-6
rKRTAP28-8
rKRTAP28-3
rKRTAP28-10
rKRTAP28-11
rKRTAP28-1
rKRTAP28-5
hKRTAP28-1
hKRTAP28-8
hKRTAP28-3
hKRTAP28-4
hKRTAP28-7
hKRTAP28-5
hKRTAP28-2
hKRTAP28-6
rKRTAP28-2
28
rKRTAP5-18
rKRTAP5-15
rKRTAP5-13
rKRTAP5-16
rKRTAP5-17
rKRTAP5-1
rKRTAP5-12
rKRTAP5-14
hKRTAP5-7
hKRTAP5-4
hKRTAP5-2
hKRTAP5-5
hKRTAP5-11
hKRTAP5-1
hKRTAP5-10
hKRTAP5-6
hKRTAP5-9
hKRTAP5-8
hKRTAP5-3
rKRTAP5-3
rKRTAP5-11
rKRTAP5-5
rKRTAP5-9
rKRTAP5-6
rKRTAP5-7
rKRTAP5-8
rKRTAP5-2
rKRTAP5-4
rKRTAP5-10
rKRTAP5-19
5
hKRTAP26-1
rKRTAP26-1
rKRTAP34-1
hKRTAP29-1
rKRTAP29-1
rKRTAP3-4
hKRTAP3-1
rKRTAP3-1
hKRTAP3-3
hKRTAP3-2
rKRTAP3-3
rKRTAP3-2
hKRTAP25-1
hKRTAP24-1
rKRTAP24-1
hKRTAP11-1
rKRTAP11-1
hKRTAP27-1
rKRTAP27-1
rKRTAP13-3
rKRTAP13-5
rKRTAP13-4
rKRTAP13-2
rKRTAP13-6
rKRTAP13-8
rKRTAP13-7
hKRTAP13-5
rKRTAP13-10
rKRTAP13-9
rKRTAP13-1
hKRTAP13-3
hKRTAP13-4
hKRTAP13-2
hKRTAP13-1
13
0.1

Figure14

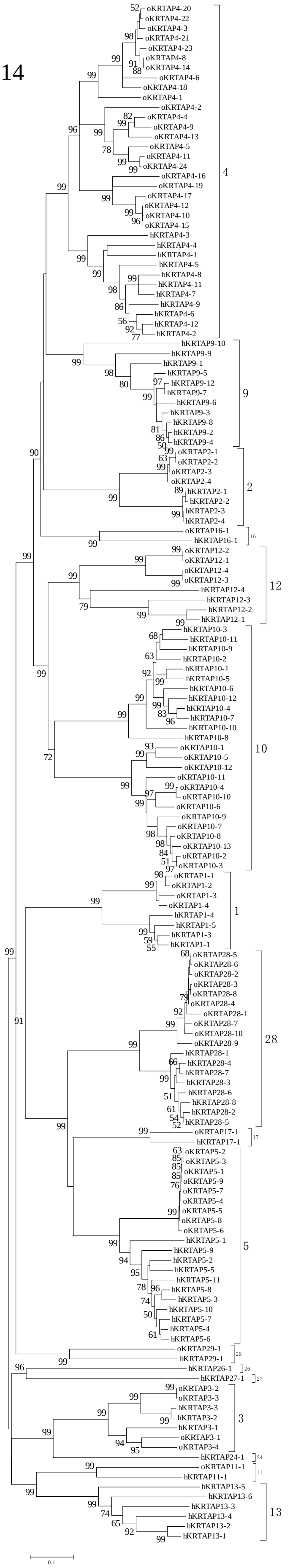

Figure15

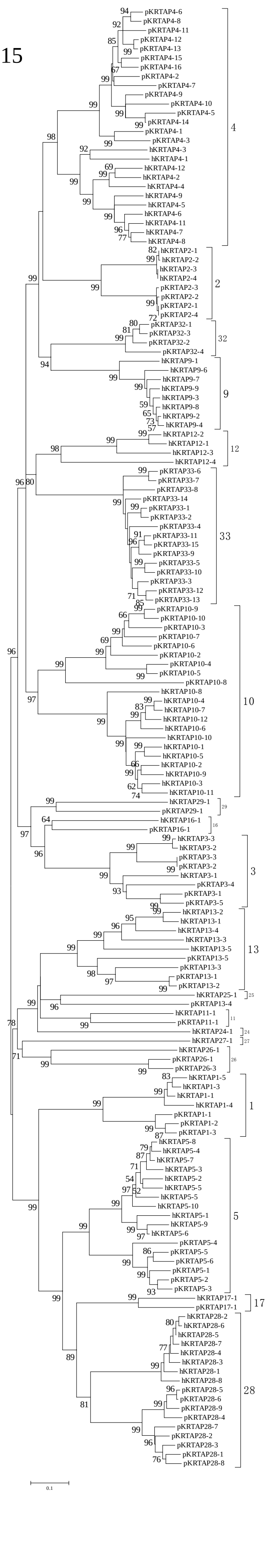
pKRTAP4-6
pKRTAP4-8
pKRTAP4-11
pKRTAP4-12
pKRTAP4-13
pKRTAP4-15
pKRTAP4-16
pKRTAP4-2
pKRTAP4-7
pKRTAP4-9
pKRTAP4-10
pKRTAP4-5
pKRTAP4-14
pKRTAP4-1
pKRTAP4-3
hKRTAP4-3
hKRTAP4-1
hKRTAP4-12
hKRTAP4-2
hKRTAP4-4
hKRTAP4-9
hKRTAP4-5
hKRTAP4-6
hKRTAP4-11
hKRTAP4-7
hKRTAP4-8
4
hKRTAP2-1
hKRTAP2-2
hKRTAP2-3
hKRTAP2-4
pKRTAP2-3
pKRTAP2-2
pKRTAP2-1
pKRTAP2-4
2
pKRTAP32-1
pKRTAP32-3
pKRTAP32-2
pKRTAP32-4
32
hKRTAP9-1
hKRTAP9-6
hKRTAP9-7
hKRTAP9-9
hKRTAP9-3
hKRTAP9-8
hKRTAP9-2
hKRTAP9-4
9
hKRTAP12-2
hKRTAP12-1
hKRTAP12-3
hKRTAP12-4
12
pKRTAP33-6
pKRTAP33-7
pKRTAP33-8
pKRTAP33-14
pKRTAP33-1
pKRTAP33-2
pKRTAP33-4
pKRTAP33-11
pKRTAP33-15
pKRTAP33-9
pKRTAP33-5
pKRTAP33-10
pKRTAP33-3
pKRTAP33-12
pKRTAP33-13
33
pKRTAP10-9
pKRTAP10-10
pKRTAP10-3
pKRTAP10-7
pKRTAP10-6
pKRTAP10-2
pKRTAP10-4
pKRTAP10-5
pKRTAP10-8
hKRTAP10-8
hKRTAP10-4
hKRTAP10-7
hKRTAP10-12
hKRTAP10-6
hKRTAP10-10
hKRTAP10-1
hKRTAP10-5
hKRTAP10-2
hKRTAP10-9
hKRTAP10-3
hKRTAP10-11
10
hKRTAP29-1
pKRTAP29-1
29
hKRTAP16-1
pKRTAP16-1
16
hKRTAP3-3
hKRTAP3-2
pKRTAP3-3
pKRTAP3-2
hKRTAP3-1
pKRTAP3-4
pKRTAP3-1
pKRTAP3-5
3
hKRTAP13-2
hKRTAP13-1
hKRTAP13-4
hKRTAP13-3
hKRTAP13-5
pKRTAP13-5
pKRTAP13-3
pKRTAP13-1
pKRTAP13-2
13
hKRTAP25-1
25
pKRTAP13-4
hKRTAP11-1
pKRTAP11-1
11
hKRTAP24-1
24
hKRTAP27-1
27
hKRTAP26-1
pKRTAP26-1
pKRTAP26-3
26
hKRTAP1-5
hKRTAP1-3
hKRTAP1-1
hKRTAP1-4
pKRTAP1-1
pKRTAP1-2
pKRTAP1-3
1
hKRTAP5-8
hKRTAP5-4
hKRTAP5-7
hKRTAP5-3
hKRTAP5-2
hKRTAP5-5
hKRTAP5-5
hKRTAP5-10
hKRTAP5-1
hKRTAP5-9
hKRTAP5-6
pKRTAP5-4
pKRTAP5-5
pKRTAP5-6
pKRTAP5-1
pKRTAP5-2
pKRTAP5-3
5
hKRTAP17-1
pKRTAP17-1
17
hKRTAP28-2
hKRTAP28-6
hKRTAP28-5
hKRTAP28-7
hKRTAP28-4
hKRTAP28-3
hKRTAP28-1
hKRTAP28-8
pKRTAP28-5
pKRTAP28-6
pKRTAP28-9
pKRTAP28-4
pKRTAP28-7
pKRTAP28-2
pKRTAP28-3
pKRTAP28-1
pKRTAP28-8
28
0.1

Figure16

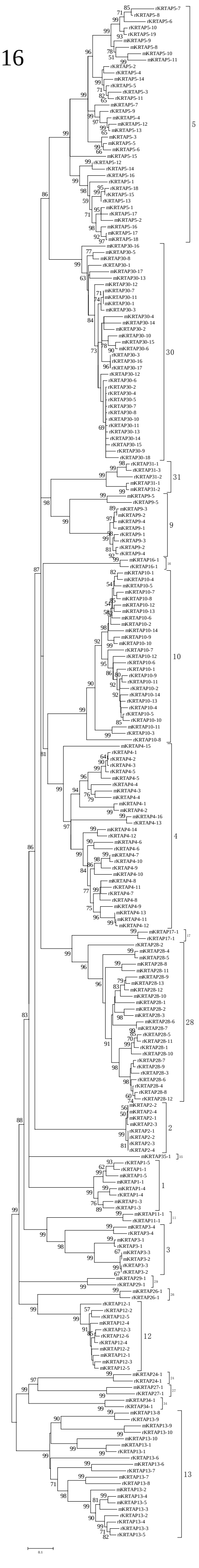

Figure17

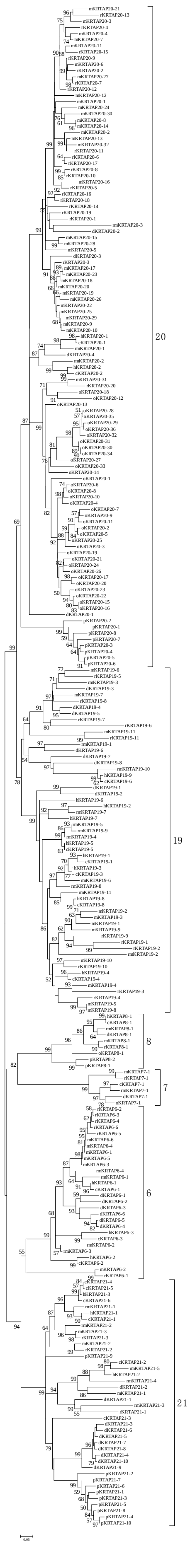

Supplement: Additional file 2 — figure 1–figure 17. Figure 1–Figure 16 are the phylogenetic trees of high/ultrahigh cysteine KAPs of human (Figure 1), chimpanzee (Figure 2), rhesus macaque(figure 3), dog (figure 4), mouse (figure 5), rat (figure 6), opossum (figure 7), platypus(figure 8), human and chimpanzee (figure 9), human and rhesus macaque (figure 10), human and dog (figure 11), human and mouse (figure 12), human and rat (figure 13), human and opossum (figure 14), human and platypus (figure 15), mouse and rat (figure 16). Figure 17 is the phylogenetic tree of high glycine/tyrosine KRTAPs. h represents human, c is chimpanzee, rh is rhesus macaque, d-dog, m-mouse, r is rat, o is opossum, and p is platypus. The values on the branches are reliabilities, which are evaluated by the interior branch tests with 1,000 replications. Only values higher than 50% are noted. [file 1471-2148-8-241-S2.pdf]
