# Supplementary material for: Molecular evolution of the keratin associated protein gene family in mammals, role in the evolution of mammalian hair
Source: BMC Evol Biol. 2008 Aug 23;8:241. doi: 10.1186/1471-2148-8-241 (PMC2528016; doi:10.1186/1471-2148-8-241)
Supplement: Additional file 3 — table 2. Pairs of genes with significant statistical support for gene conversion. The significances are calculated by Bonferroni-corrected method. [file 1471-2148-8-241-S3.doc]

|  | Gene pairs | Length | P-values |
| --- | --- | --- | --- |
| human | KRTAP10-3: KRTAP10-10 | 50 | 0.0135 |
|  | KRTAP10-5: KRTAP10-10 | 50 | 0.01839 |
|  | KRTAP10-2: KRTAP10-5 | 78 | 0.03291 |
|  | KRTAP10-2: KRTAP10-3 | 78 | 0.04805 |
|  | KRTAP13-2: KRTAP13-3 | 76 | 0.00301 |
|  | KRTAP13-6: KRTAP13-3 | 24 | 0.00488 |
| chimpanzee | KRTAP3-4: KRTAP3-1 | 25 | 0.00013 |
|  | KRTAP5-3: KRTAP5-5 | 50 | 0.03802 |
|  | KRTAP13-2: KRTAP13-5 | 20 | 0.00162 |
|  | KRTAP13-1: KRTAP13-5 | 20 | 0.00466 |
|  | KRTAP10-3: KRTAP10-12 | 131 | 0.0003 |
|  | KRTAP10-12: KRTAP10-13 | 110 | 0.00047 |
|  | KRTAP10-5: KRTAP10-12 | 83 | 0.00762 |
|  | KRTAP10-11: KRTAP10-12 | 98 | 0.00977 |
|  | KRTAP10-4: KRTAP10-8 | 69 | 0.01593 |
|  | KRTAP10-7: KRTAP10-12 | 101 | 0.02247 |
|  | KRTAP10-10: KRTAP10-12 | 42 | 0.03594 |
|  | KRTAP10-5: KRTAP10-6 | 59 | 0.04198 |
| rhesus macaque | KRTAP4-4: KRTAP4-3 | 120 | 0.00002 |
|  | KRTAP9-1:KRTAP9-2 | 20 | 0.0182 |
|  | KRTAP9-1:KRTAP9-4 | 332 | 0.03476 |
|  | KRTAP9-1:KRTAP9-4 | 20 | 0.03476 |
|  | KRTAP12-2: KRTAP12-4 | 26 | 0.04779 |
| mouse | KRTAP3-3: KRTAP3-1 | 98 | 0.00026 |
|  | KRTAP3-2: KRTAP3-1 | 98 | 0.00026 |
|  | KRTAP3-1: KRTAP3-4 | 13 | 0.02178 |
|  | KRTAP9-3: KRTAP9-5 | 13 | 0.00181 |
|  | KRTAP9-2: KRTAP9-5 | 13 | 0.00385 |
|  | KRTAP9-3: KRTAP9-4 | 566 | 0.00467 |
|  | KRTAP5-6: KRTAP5-15 | 30 | <0.00001 |
|  | KRTAP5-6:KRTAP5-16 | 30 | <0.00001 |
|  | KRTAP5-6: KRTAP5-17 | 30 | <0.00001 |
|  | KRTAP5-6: KRTAP5-18 | 30 | <0.00001 |
|  | KRTAP5-6: KRTAP5-1 | 30 | <0.00001 |
|  | KRTAP5-9: KRTAP5-4 | 31 | 0.00543 |
|  | KRTAP5-15: KRTAP5-4 | 27 | 0.01864 |
|  | KRTAP5-16: KRTAP5-4 | 27 | 0.01864 |
|  | KRTAP5-9: KRTAP5-12 | 31 | 0.02394 |
|  | KRTAP5-17: KRTAP5-4 | 27 | 0.02495 |
|  | KRTAP5-18: KRTAP5-4 | 27 | 0.02495 |
|  | KRTAP5-1: KRTAP5-4 | 27 | 0.02495 |
|  | KRTAP13-2: KRTAP13-5 | 160 | <0.00001 |
|  | KRTAP13-2: KRTAP13-4 | 160 | <0.00001 |
|  | KRTAP13-2: KRTAP13-3 | 112 | 0.00002 |
|  | KRTAP13-3: KRTAP13-4 | 117 | 0.00027 |
|  | KRTAP13-4: KRTAP13-5 | 165 | 0.001 |
|  | KRTAP13-3: KRTAP13-5 | 117 | 0.00294 |
|  | KRTAP13-2: KRTAP13-7 | 39 | 0.04864 |
|  | KRTAP10-5: KRTAP10-11 | 72 | 0.00142 |
|  | KRTAP10-7: KRTAP10-11 | 83 | 0.00229 |
|  | KRTAP10-11: KRTAP10-13 | 85 | 0.00293 |
|  | KRTAP10-8: KRTAP10-11 | 72 | 0.00335 |
|  | KRTAP10-10: KRTAP10-14 | 44 | 0.00348 |
|  | KRTAP10-9: KRTAP10-11 | 94 | 0.0043 |
|  | KRTAP10-10: KRTAP10-11 | 85 | 0.01213 |
|  | KRTAP21-1: KRTAP21-2 | 80 | 0.04146 |
| rat | KRTAP4-9: KRTAP4-12 | 49 | 0.03024 |
|  | KRTAP4-2: KRTAP4-6 | 89 | 0.03779 |
|  | KRTAP9-1: KRTAP9-5 | 26 | 0.0187 |
|  | KRTAP9-1: KRTAP9-4 | 244 | 0.01887 |
|  | KRTAP5-6: KRTAP5-7 | 95 | 0.00074 |
|  | KRTAP5-5: KRTAP5-16 | 35 | 0.04517 |
|  | KRTAP5-9: KRTAP5-16 | 35 | 0.04517 |
|  | KRTAP5-2: KRTAP5-7 | 82 | 0.04524 |
|  | KRTAP13-2: KRTAP13-3 | 138 | <0.00001 |
|  | KRTAP13-2: KRTAP13-4 | 89 | 0.00225 |
|  | KRTAP13-2: KRTAP13-5 | 89 | 0.00655 |
|  | KRTAP13-4: KRTAP13-5 | 138 | 0.00255 |
|  | KRTAP13-2: KRTAP13-4 | 48 | 0.01327 |
|  | KRTAP13-2: KRTAP13-5 | 48 | 0.0333 |
|  | KRTAP10-3: KRTAP10-4 | 116 | <0.00001 |
|  | KRTAP10-3: KRTAP10-9 | 116 | <0.00001 |
|  | KRTAP10-8: KRTAP10-12 | 64 | 0.00001 |
|  | KRTAP10-6: KRTAP10-8 | 64 | 0.00051 |
|  | KRTAP10-3: KRTAP10-11 | 115 | 0.0011 |
|  | KRTAP10-8: KRTAP10-14 | 64 | 0.00163 |
|  | KRTAP10-3: KRTAP10-7 | 80 | 0.00181 |
|  | KRTAP10-7: KRTAP10-8 | 52 | 0.00188 |
|  | KRTAP10-6: KRTAP10-12 | 72 | 0.00265 |
|  | KRTAP10-1: KRTAP10-12 | 72 | 0.00393 |
|  | KRTAP10-2: KRTAP10-12 | 72 | 0.00579 |
|  | KRTAP10-5: KRTAP10-12 | 72 | 0.00579 |
|  | KRTAP10-8: KRTAP10-10 | 72 | 0.00579 |
|  | KRTAP10-11: KRTAP10-12 | 72 | 0.00579 |
|  | KRTAP10-8: KRTAP10-9 | 96 | 0.00921 |
|  | KRTAP10-5: KRTAP10-8 | 64 | 0.01019 |
|  | KRTAP10-1: KRTAP10-8 | 64 | 0.0145 |
|  | KRTAP10-8: KRTAP10-11 | 64 | 0.0145 |
|  | KRTAP10-10: KRTAP10-12 | 88 | 0.01497 |
|  | KRTAP10-12: KRTAP10-14 | 72 | 0.01802 |
|  | KRTAP10-3: KRTAP10-5 | 76 | 0.01889 |
|  | KRTAP10-3: KRTAP10-10 | 40 | 0.01981 |
|  | KRTAP10-2: KRTAP10-8 | 64 | 0.02055 |
|  | KRTAP10-8: KRTAP10-13 | 64 | 0.02055 |
|  | KRTAP10-9: KRTAP10-12 | 64 | 0.02055 |
|  | KRTAP10-6: KRTAP10-14 | 72 | 0.02606 |
|  | KRTAP10-12: KRTAP10-13 | 72 | 0.02606 |
|  | KRTAP10-3: KRTAP10-12 | 67 | 0.02784 |
|  | KRTAP10-1: KRTAP10-3 | 75 | 0.03181 |
|  | KRTAP10-1: KRTAP10-3 | 40 | 0.03181 |
|  | KRTAP10-2: KRTAP10-3 | 75 | 0.04007 |
|  | KRTAP10-2: KRTAP10-3 | 40 | 0.04007 |
|  | KRTAP12-3: KRTAP12-6 | 110 | 0.00503 |
|  | KRTAP31-3: KRTAP31-2 | 149 | 0.04915 |
|  | KRTAP19-6: KRTAP19-7 | 127 | 0.02715 |
| dog | KRTAP3-1: KRTAP3-4 | 29 | 0.00529 |
|  | KRTAP4-7: KRTAP4-3 | 83 | 0.00009 |
|  | KRTAP4-7: KRTAP4-2 | 83 | 0.00009 |
|  | KRTAP4-5: KRTAP4-3 | 24 | 0.00021 |
|  | KRTAP4-5: KRTAP4-2 | 24 | 0.00021 |
|  | KRTAP4-5: KRTAP4-4 | 57 | 0.00065 |
|  | KRTAP4-8: KRTAP4-6 | 341 | 0.01333 |
|  | KRTAP4-6: KRTAP4-3 | 26 | 0.02204 |
|  | KRTAP4-6: KRTAP4-2 | 26 | 0.02204 |
|  | KRTAP4-8: KRTAP4-5 | 225 | 0.04981 |
|  | KRTAP13-1: KRTAP13-5 | 49 | 0.00488 |
|  | KRTAP13-1: KRTAP13-3 | 72 | 0.02984 |
|  | KRTAP10-5: KRTAP10-6 | 77 | 0.03655 |
|  | KRTAP12-1: KRTAP12-6 | 65 | <0.00001 |
|  | KRTAP12-6: KRTAP12-4 | 65 | 0.00001 |
|  | KRTAP12-6: KRTAP12-7 | 65 | 0.00001 |
|  | KRTAP12-1: KRTAP12-2 | 58 | 0.00162 |
|  | KRTAP12-2:KRTAP12-4 | 58 | 0.00162 |
|  | KRTAP12-2: KRTAP12-7 | 58 | 0.00162 |
|  | KRTAP12-1: KRTAP12-3 | 14 | 0.00868 |
|  | KRTAP12-3: KRTAP12-4 | 14 | 0.01465 |
|  | KRTAP12-3: KRTAP12-7 | 14 | 0.01465 |
|  | KRTAP28-1: KRTAP28-5 | 45 | 0.04209 |
| opossum | KRTAP3-1: KRTAP3-4 | 43 | 0.02842 |
|  | KRTAP10-3: KRTAP10-12 | 88 | 0.00971 |
|  | KRTAP10-2:KRTAP10-12 | 88 | 0.0146 |
|  | KRTAP10-7: KRTAP10-9 | 198 | 0.03175 |
|  | KRTAP4-3: KRTAP4-10 | 37 | 0.00752 |
|  | KRTAP4-3: KRTAP4-12 | 37 | 0.00752 |
|  | KRTAP4-3: KRTAP4-15 | 37 | 0.00752 |
|  | KRTAP4-10: KRTAP4-14 | 37 | 0.01628 |
|  | KRTAP4-12: KRTAP4-14 | 37 | 0.01628 |
|  | KRTAP4-14: KRTAP4-15 | 37 | 0.01628 |
|  | KRTAP28-3: KRTAP28-8 | 134 | 0.03617 |
| platypus | KRTAP3-1: KRTAP3-5 | 148 | 0.00814 |
|  | KRTAP3-1: KRTAP3-5 | 94 | 0.02807 |
|  | KRTAP5-2: KRTAP5-3 | 45 | <0.00001 |
|  | KRTAP5-1: KRTAP5-3 | 37 | <0.00001 |
|  | KRTAP5-1: KRTAP5-3 | 32 | <0.00001 |
|  | KRTAP5-1: KRTAP5-6 | 41 | 0.00002 |
|  | KRTAP5-2: KRTAP5-6 | 26 | 0.00009 |
|  | KRTAP5-1: KRTAP5-2 | 35 | 0.00009 |
|  | KRTAP5-3: KRTAP5-6 | 31 | 0.00011 |
|  | KRTAP5-1: KRTAP5-2 | 33 | 0.00029 |
|  | KRTAP5-3: KRTAP5-6 | 27 | 0.00046 |
|  | KRTAP5-1: KRTAP5-2 | 26 | 0.00292 |
|  | KRTAP5-2: KRTAP5-3 | 29 | 0.00317 |
|  | KRTAP5-2: KRTAP5-6 | 20 | 0.00401 |
|  | KRTAP5-1: KRTAP5-3 | 17 | 0.0042 |
|  | KRTAP5-1: KRTAP5-2 | 26 | 0.00522 |
|  | KRTAP5-2: KRTAP5-6 | 21 | 0.01859 |
|  | KRTAP5-2: KRTAP5-3 | 22 | 0.03182 |
|  | KRTAP5-2: KRTAP5-3 | 22 | 0.03182 |
|  | KRTAP10-4: KRTAP10-5 | 473 | 0.0001 |
|  | KRTAP21-3: KRTAP21-8 | 75 | 0.00050 |
|  | KRTAP21-2: KRTAP21-8 | 25 | 0.04274 |
