# Supplementary material for: Molecular evolution of the keratin associated protein gene family in mammals, role in the evolution of mammalian hair
Source: BMC Evol Biol. 2008 Aug 23;8:241. doi: 10.1186/1471-2148-8-241 (PMC2528016; doi:10.1186/1471-2148-8-241)
Supplement: Additional file 4 — table 3. Results of recombination detection by RDP2 program with algorithms: RDP, BootScan, MaxChi and Chimaera with 1,000 permutations. Sequences were considered linear. The highest acceptable P value cut-off was set to 0.01, and the Bonferroni correction was employed. The numbers are the unique events (recombination signals). [file 1471-2148-8-241-S4.doc]

| subfamily | human | chimpanzee | rhesus macaque | dog | mouse | rat | opossum | platypus |
| --- | --- | --- | --- | --- | --- | --- | --- | --- |
| 1 | 1(1) | 0(0) | 0(0) | 0(0) | 0(0) | 0(0) | 1(4) | 0(0) |
| 2 | 0(0) | 0(0) | 0(0) | 0(0) |  | 0(0) | 0(0) | 0(0) |
| 3 | 0(0) | 1(3) | 0(0) | 1(3) | 1(6) | 0(0) | 1(4) | 1(4) |
| 4 | 3(11) | 4(17) | 5(19) | 2(14) | 16(24) | 3(15) | 10(53) | 5(16) |
| 5 | 1(0) | 6(12) | 5(9) | 3(3) | 11(33) | 12(72) | 0(0) | 2(3) |
| 9 | 3(5) | 0(0) | 0(0) | 1(2) | 4(6) | 1(3) |  |  |
| 10 | 3(12) | 5(11) | 4(12) | 3(10) | 5(12) | 4(13) | 6(14) | 10(33) |
| 12 | 1(2) | 0(0) | 1(2) | 1(4) | 0(0) | 2(4) | 0(0) |  |
| 13 | 2(8) | 1(3) | 1(1) | 4(5) | 6(15) | 4(11) |  | 1(1) |
| 28 | 0(0) | 1(2) | 1(1) | 3(7) | 3(4) | 1(6) | 2(4) | 1(2) |
| 30 |  |  |  |  | 0(0) | 0(0) |  |  |
| 31 |  |  |  |  |  | 0(0) |  |  |
| 32 |  |  |  |  |  |  |  | 0(0) |
| 33 |  |  |  |  |  |  |  | 4(10) |
| 6 | 0(0) | 0(0) | 0(0) | 0(0) | 0(0) | 0(0) |  |  |
| 19 | 0(0) | 0(0) | 0(0) | 0(0) | 0(0) | 1(4) |  |  |
| 20 |  |  | 0(0) | 0(0) | 0(0 | 1(0) | 0(0) | 0(0) |
| 21 | 1(1) | 1(1) | 0(0) | 0(0) | 1(4) | 0(0) |  | 2(6) |
